# mDia1 Assembles a Linear F-Actin Coat at Membrane Invaginations To Drive Listeria monocytogenes Cell-to-Cell Spreading

**DOI:** 10.1128/mBio.02939-21

**Published:** 2021-11-16

**Authors:** Aaron S. Dhanda, A. Wayne Vogl, Fern Ness, Metello Innocenti, Julian A. Guttman

**Affiliations:** a Department of Biological Sciences, Centre for Cell Biology, Development, and Disease, Simon Fraser University, Burnaby, British Columbia, Canada; b Life Sciences Institute and Department of Cellular and Physiological Sciences, Faculty of Medicine, University of British Columbia, Vancouver, British Columbia, Canada; c Heidelberg University Biochemistry Center (BZH), Heidelberg University, Heidelberg, Germany; University of Washington

**Keywords:** *Listeria monocytogenes*, actin, cytoskeleton, endocytosis, host-pathogen interactions

## Abstract

Direct cell-to-cell spreading of Listeria monocytogenes requires the bacteria to induce actin-based finger-like membrane protrusions in donor host cells that are endocytosed through caveolin-rich membrane invaginations by adjacent receiving cells. An actin shell surrounds these endocytic sites; however, its structure, composition, and functional significance remain elusive. Here, we show that the formin mDia1, but surprisingly not the Arp2/3 complex, is enriched at the membrane invaginations generated by L. monocytogenes during HeLa and Jeg-3 cell infections. Electron microscopy reveals a band of linear actin filaments that run along the longitudinal axis of the invagination membrane. Mechanistically, mDia1 expression is vital for the assembly of this F-actin shell. mDia1 is also required for the recruitment of Filamin A, a caveola-associated F-actin cross-linking protein, and caveolin-1 to the invaginations. Importantly, mixed-cell infection assays show that optimal caveolin-based L. monocytogenes cell-to-cell spreading correlates with the formation of the linear actin filament-containing shell by mDia1.

## INTRODUCTION

Metazoan cellular motility and endocytosis rely on the regulated assembly and dynamic organization of actin filaments (F-actin) into branched and linear networks (see references 1 to 3 for reviews). The bacterial pathogen Listeria monocytogenes routinely hijacks these actin arrays during its own infections as it invades host cells (see reference 4 for a review), moves within them (see references 5 and 6 for reviews), and ultimately disseminates between them (see reference 7 for a review). The resulting human clinical syndromes of L. monocytogenes infection are vast and diverse and generally arise from the bacteria targeting a variety of host cell types and tissues ranging from epithelial and myeloid cells found within the intestine, liver, brain, and placenta to the microvascular endothelial cells that permeate these organs (8).

To initially enter nonphagocytic host epithelial cells, L. monocytogenes hijacks the clathrin-mediated endocytic (CME) machinery (9–11) and as part of this process, exploits classical CME and actin cytoskeletal components, including the Arp2/3 complex, to rapidly generate a branched actin-containing endocytic cup (9–17). Once internalized, the bacteria escape a membrane-containing vacuole to gain access to the host cell cytoplasm. There, L. monocytogenes bacteria utilize their surface-bound effector protein ActA (18, 19) to recruit and activate the Arp2/3 complex, thereby giving rise to a branched actin filament network that allows the bacteria to move both intracellularly and intercellularly via comet/rocket tails and membrane protrusions, respectively (20, 21). Although the Arp2/3 complex is largely responsible for generating the branched F-actin network of cytoplasmic comet/rocket tails (16, 20–22), once motile bacteria engage with the host cell periphery to initiate cell-to-cell spreading, the formin class of actin nucleating proteins also become involved to incorporate linear actin cables within the protrusions (23, 24). This network of branched and unbranched actin filaments (23, 24) helps to efficiently propel the bacteria against the host cell plasma membrane as it distends the membrane outwards into a long bacterium-led finger-like membrane protrusion. This protrusion pushes against the surface of an adjacent host cell, producing a corresponding membrane invagination that endocytoses the micrometer-scale bacterium-containing structure through a caveolin-dependent mechanism (25, 26). A requirement for successful L. monocytogenes infections in host organs is the ability of the bacteria to undergo cell-to-cell spreading as infections (both *in vitro* and *in vivo*) with mutant bacteria that are defective in generating membrane protrusions/invaginations are nonfatal and are often characterized by significantly diminished organ colonization (19, 27–31). Interestingly, we recently reported on the existence of a thin F-actin shell closely surrounding these pathologically vital bacterially induced membrane invaginations (26). However, its structural organization and functional significance in the context of L. monocytogenes infections are not known.

Here, we set out to investigate how and why such an actin shell surrounds L. monocytogenes membrane invaginations during the cell-to-cell dissemination of the bacteria. Using light and electron microscopy, infection assays and loss-of-function approaches, we show that these large endocytic structures are coated by parallel linear actin filaments assembled by the formin mDia1. These membrane invaginations contain the caveola-associated F-actin-binding proteins Filamin A and myosin 1c (Myo1c). Importantly, proper formation of an mDia1-nucleated F-actin coat is crucial for the recruitment of Filamin A and caveolin-1 and for optimal cell-to-cell spreading of L. monocytogenes. Taken together, our findings demonstrate a new role for formins and linear actin filaments during noncanonical micro-sized caveolin-based endocytic events and shed new light on the molecular mechanisms driving L. monocytogenes intercellular dissemination.

## RESULTS

### mDia1 but not the Arp2/3 complex is enriched along with F-actin at membrane invaginations induced by L. monocytogenes.

**FIG 1:**
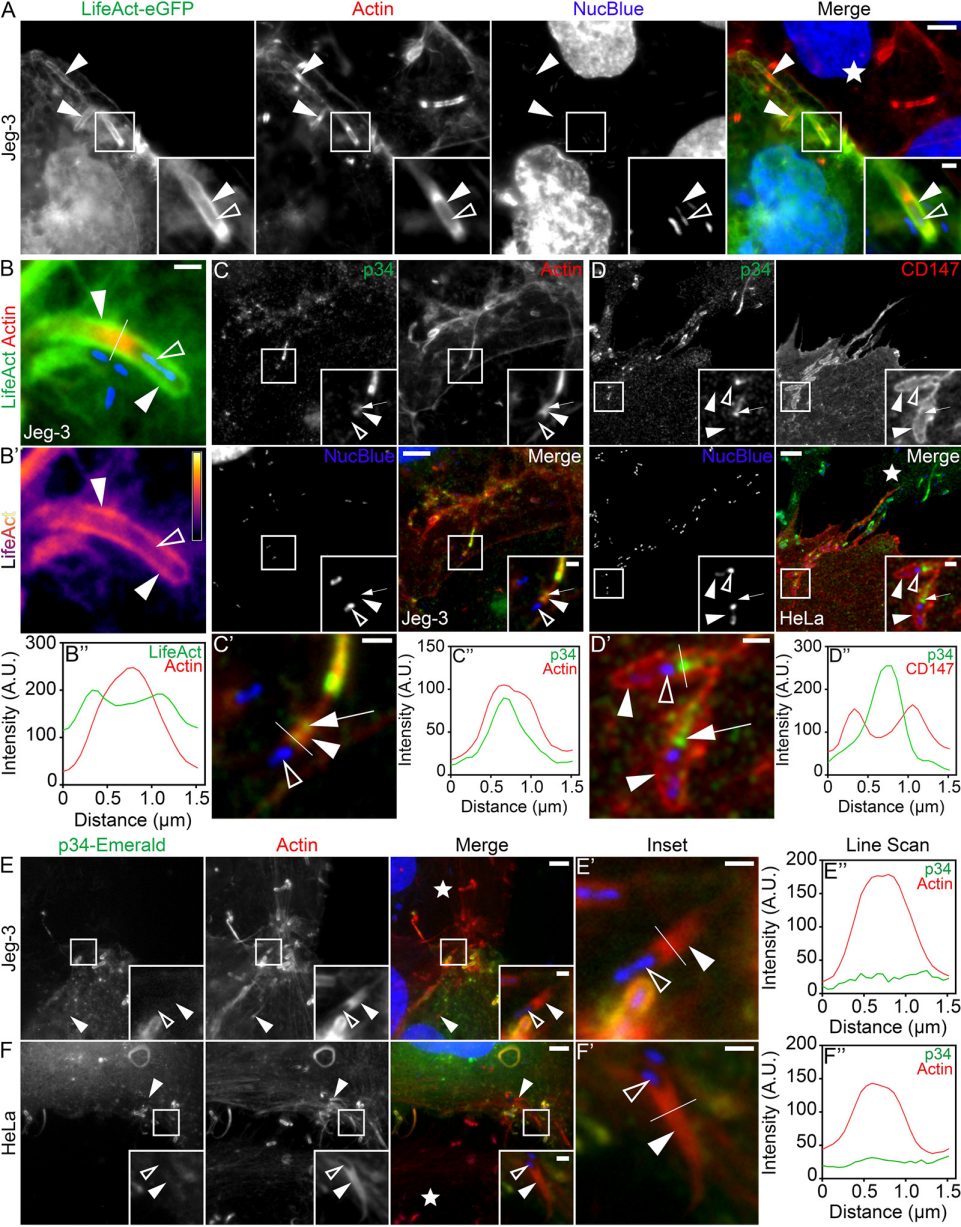
The Arp2/3 complex does not localize to L. monocytogenes actin-rich membrane invaginations. (A) Mixed Jeg-3 cell assay showing LifeAct-eGFP (green) concentrated at bacterial membrane invaginations. Alexa Fluor 594-phalloidin (indicated as “Actin”; red) was used to visualize total F-actin (in both the protrusion-forming and invagination-forming cells) and NucBlue (blue) to visualize host DNA in addition to bacteria present within the invaginations. Bars, 5 μm and 1 μm (insets). (B to B’’) Line scan analysis of an L. monocytogenes membrane protrusion/invagination from samples stained with fluorescent phalloidin (indicated as “Actin”; red) to visualize total F-actin and NucBlue (blue) to confirm the presence of the bacteria at the structures. Intensity is shown in arbitrary units (A.U.). (B’) A heat map of the representative LifeAct signal from panel B (light yellow indicates the highest signal intensity). Bar, 1 μm. (B’’) A 1.5-μm line (white line) was drawn through the protrusion/invagination (see panel B). The total F-actin intensity (red) as well as the corresponding LifeAct intensity (green) was plotted. (C) Jeg-3 cells were infected with L. monocytogenes, fixed, and stained with p34/ArpC2 targeting antibodies (green), NucBlue (blue) to visualize host cell DNA and bacteria, and Alexa Fluor 594-phalloidin (red) to visualize F-actin. Insets shown are an enlargement of the boxed regions. Bars, 5 μm and 1 μm (inset). (C’ and C’’) Line scan analysis of the L. monocytogenes membrane protrusion/invagination from the merge inset in panel C. (C’) Enlargement of the inset shown in panel C. Bar, 1 μm. (C’’) A 1.5-μm line (white line) was drawn through the protrusion/invagination in panel C’, and the total F-actin intensity (red) as well as the corresponding p34/Arpc2 intensity (green) was plotted. (D) Mixed HeLa cell assay demonstrating that endogenous p34/ArpC2 (green) concentrates in the actin-rich core of bacterial membrane protrusions but not at membrane invaginations. The membrane invagination marker CD147-GFP (pseudo-colored red) is expressed solely in the membrane invagination-forming host cell. Samples were fixed and stained with NucBlue (blue) to visualize host DNA as well as any bacteria within the invaginations. Bars, 5 μm and 1 μm (inset). (D’ and D’’) Line scan analysis of the L. monocytogenes membrane protrusion/invagination from the merge inset in panel D. (D’) Enlargement of the inset in panel D. Bar, 1 μm. (D’’) A 1.5-μm line (white line) was drawn through the protrusion/invagination in panel D’, and the CD147-GFP intensity (red) as well as the corresponding p34/Arpc2 intensity (green) was plotted. (E) Mixed Jeg-3 cell assay demonstrating p34-Emerald (green) absence at L. monocytogenes membrane invaginations when expressed in the invagination-forming cells, Alexa Fluor 594-phalloidin (red) to visualize F-actin and NucBlue (blue) to visualize host DNA and bacteria within the invaginations. Bars, 5 μm or 1 μm (inset). (E’ and E’’) Line scan analysis of the L. monocytogenes membrane protrusion/invagination from the merge inset in panel E. (E’) Enlargement of the merge inset shown in panel E. Bar, 1 μm. (C’’) A 1.5-μm line (white line) was drawn through the protrusion/invagination in panel E’, and the total F-actin intensity (red) as well as the corresponding p34-Emerald intensity (green) was plotted. (F to F’’) The same as shown in panels E to E’’ but using HeLa cells in place of Jeg-3 cells. The white stars in the merge panels indicate the locations of the untransfected protrusion-forming host cells. All insets are enlargements of the boxed regions. Color intensities are enhanced in the insets to clearly visualize the labeled proteins. Solid arrowheads indicate the invagination, and open arrowheads indicate the spreading bacteria. Full arrows point to p34/ArpC2 present within the membrane protrusion actin-rich core.

F-actin has been shown to surround L. monocytogenes membrane invaginations in HeLa cells (26). As cell-to-cell spreading is crucial for placental colonization by L. monocytogenes (27, 28, 30, 32; see also reference 33 for a review), we decided to confirm these findings in Jeg-3 cells, a routinely utilized placental epithelial cell line (34–36). To do this, we used mixed-cell assays (26, 36–38) whereby infected Jeg-3 cells were overlaid onto uninfected cells expressing the F-actin-binding peptide LifeAct-eGFP (enhanced green fluorescent protein). Under these conditions, LifeAct clearly outlined the membrane invaginations present at the L. monocytogenes secondary infection sites in the recipient cells (Fig. 1A). These results support that the formation of an F-actin shell is not a cell line-specific phenomenon. To characterize this F-actin accumulation in more detail, we plotted the intensity profiles of LifeAct and phalloidin (which label either F-actin in the invagination-forming cell only or F-actin in both the protrusion-forming and invagination-forming cells, respectively) by drawing a line perpendicularly across the membrane protrusion/invagination. These analyses revealed two LifeAct peaks, indicative of LifeAct at the membrane invagination, surrounding a single phalloidin peak, originating from the actin-rich core of the membrane protrusion (Fig. 1B and B’’; see also Fig. S1A to S1D’’ in the supplemental material). Heat maps also showed an increase in LifeAct signal intensity at the membrane invaginations compared with the surrounding cytoplasm (Fig. 1B’).

10.1128/mBio.02939-21.1 FIG S1 Additional characterization of fluorescent LifeAct and p34-Emerald at L. monocytogenes membrane invaginations. (A to D’’) Mixed Jeg-3 (A to B’’) and HeLa (C to D’’) cell assays demonstrating LifeAct-eGFP (green) concentrated at bacterial membrane invaginations when expressed in the invagination-forming host cells. Samples were stained with Alexa Fluor 594-phalloidin (red) to visualize total F-actin and NucBlue (blue) to visualize host DNA as well as any bacteria within the invaginations. The white stars indicate the locations of the untransfected protrusion-forming host cells. (A’, B’, C’, and D’) Zoomed-in regions from the corresponding boxed images in panels A, B, C and D. Color intensities are enhanced in the zoomed-in images to clearly visualize the localized proteins. Solid arrowheads indicate the invaginations, and open arrowheads indicate spreading bacteria. A 1.5-μm white line was drawn through the area of the invagination/protrusion for pixel intensity profiling. (A”, B”, C”, and D’’) The corresponding pixel intensity plots from the white line in panels A’, B’, C’, and D’. Bars are 5 μm and 2 μm (inset). (E to H’’) Mixed Jeg-3 (E to F’’) and HeLa (G to H’’) cell assays demonstrating p34(ArpC2)-Emerald (green) absence at bacterial membrane invaginations when expressed in solely in the invagination-forming host cells. Samples were stained with Alexa Fluor 594-phalloidin (red) to visualize total F-actin and NucBlue (blue) to visualize host DNA as well as any bacteria within the invaginations. The white stars indicate the location sof the untransfected protrusion-forming host cells. (E’, F’, G’, and H’) Zoomed-in regions from the corresponding boxed images in panels E, F, G and H. Color intensities are enhanced in the zoomed-in images to clearly visualize the localized proteins. Solid arrowheads indicate the invaginations, and open arrowheads indicate spreading bacteria. A 1.5-μm white line was drawn through the area of the invagination/protrusion for pixel intensity profiling. (E”, F”, G”, and H’’) The corresponding pixel intensity plots from the white line in panels E’, F’, G’, and H’. Bars are 5 μm and 2 μm (inset). (I) An expanded image from Fig. 1F depicting that p34(ArpC2)-Emerald (green) localizes correctly to actin clouds (pink box), comet/rocket tails (blue box), and membrane protrusions (orange box) when expressed in the invagination-forming host cell. Color intensities are enhanced in the zoomed-in images to clearly visualize the localized proteins. Solid arrowheads indicate p34-Emerald localization at the bacterial actin-rich structures, while the open arrowheads indicate the associated bacteria at the corresponding structures. Scale bars are 5 μm, 2 μm (orange box inset), and 1 μm (pink and blue box insets). Download FIG S1, TIF file, 2.0 MB. Copyright © 2021 Dhanda et al. 2021 Dhanda et al. https://creativecommons.org/licenses/by/4.0/ This content is distributed under the terms of the Creative Commons Attribution 4.0 International license.

Rearrangement of the eukaryotic plasma membrane during large-scale and small-scale endocytic processes is largely facilitated by branched actin filament arrays nucleated by the Arp2/3 complex 9 (39–42; see also reference 2 for a review). Thus, we next set out to immunolocalize the Arp2/3 complex (specifically targeting the ARPC2/p34 subunit) at sites of L. monocytogenes intercellular spread. Surprisingly, we found no obvious enrichment of endogenous ARPC2/p34 around the L. monocytogenes-induced membrane invaginations during Jeg-3 cell infections (Fig. 1C). Rather, line scan analysis showed ARPC2/p34 concentrated within the actin-rich core of the membrane protrusions (Fig. 1C’ and C’’). The same also held true in HeLa cells where endogenous ARPC2/p34 failed to localize at membrane invaginations labeled with the L. monocytogenes membrane invagination marker CD147-GFP (37) (Fig. 1D to D’’). To corroborate these observations, we utilized the mixed-cell assay. Again, we found no obvious enrichment of Emerald GFP-tagged ARPC2/p34 (p34-Emerald) at L. monocytogenes-induced membrane invaginations when p34-Emerald was expressed in membrane invagination-forming Jeg-3 cells (Fig. 1E). In keeping with this, line scan analysis of p34-Emerald at the area of the membrane invaginations produced a low nondescript level of signal across the entire scanned area (Fig. 1E’ and E’’ and Fig. S1E to S1F’’). Similar results were also obtained during HeLa cell infections (Fig. 1F to F’’ and Fig. S1G to S1H’’), which ensured that our findings were not cell type specific. The expected localization of p34-Emerald at other L. monocytogenes actin-rich structures such as actin clouds, cytoplasmic comet/rocket tails, and membrane protrusions in the invagination-forming cells also confirmed that p34-Emerald was expressed and localized properly (Fig. S1I).

**FIG 2:**
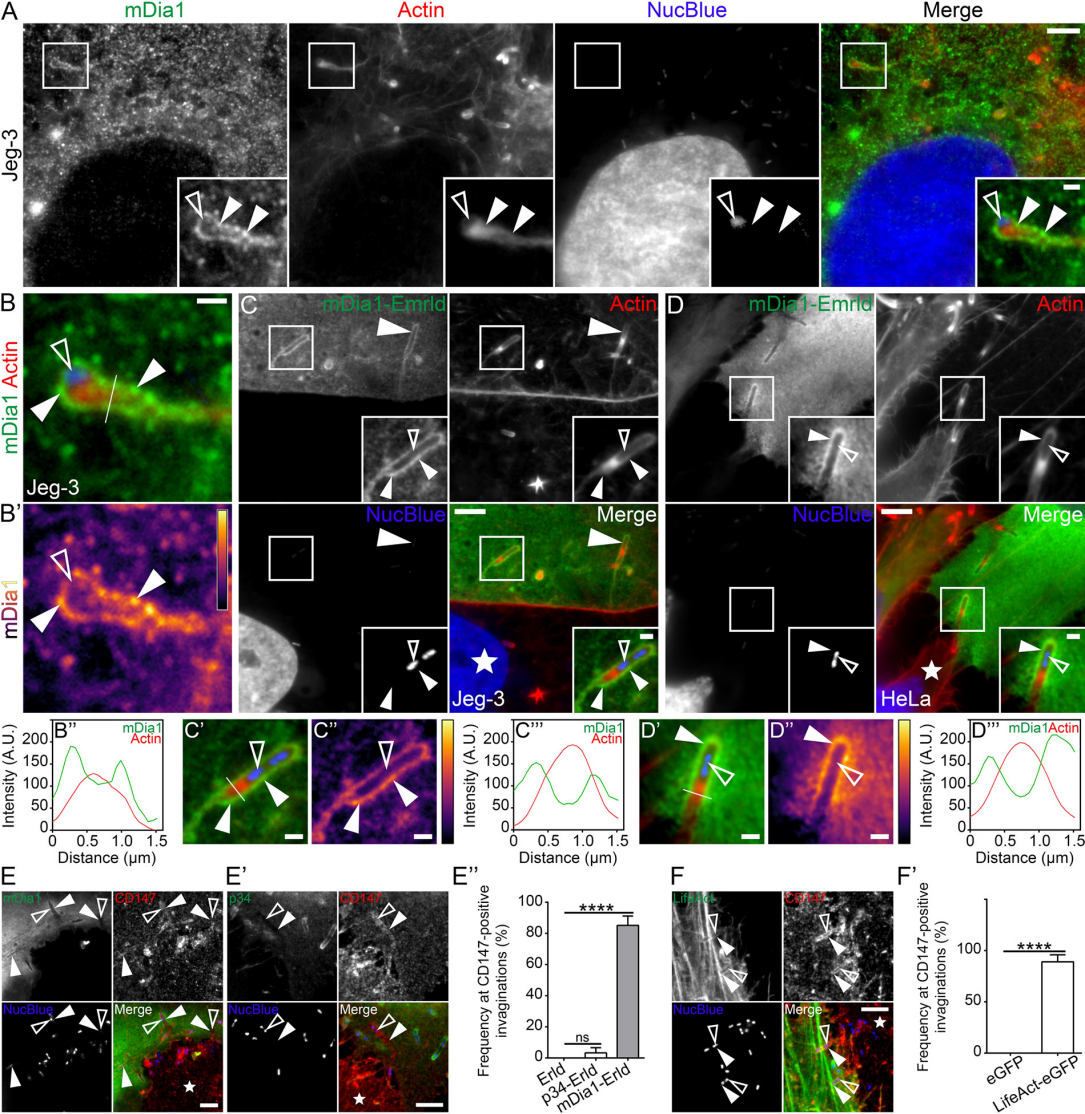
The formin mDia1 and linear actin filaments generate an actin shell around L. monocytogenes membrane invaginations. (A) Jeg-3 cells were infected with L. monocytogenes and stained with mDia1-targeting antibodies (green), NucBlue (blue) to visualize host cell DNA and bacteria, and Alexa Fluor 594-phalloidin (red) to visualize F-actin. Insets shown are an enlargement of the boxed regions. Bars, 5 μm and 1 μm (inset). (B to B’’) Line scan analysis of the L. monocytogenes membrane protrusion/invagination from the merge inset in panel A. (B) Enlargement of the merge inset in panel A. (B’) A heat map of the representative mDia1 signal from panel B (light yellow indicates the highest signal intensity). Bar, 1 μm. (B’’) A 1.5-μm line (white line) was drawn through the protrusion/invagination (see panel B) and the total F-actin intensity (red) as well as the corresponding mDia1 intensity (green) was plotted. (C) Mixed Jeg-3 cell assay demonstrating mDia1-Emerald (green) concentrated at L. monocytogenes membrane invaginations when expressed in the invagination-forming host cell. Samples were stained with Alexa Fluor 594-phalloidin (red) to visualize actin and NucBlue (blue) to visualize host DNA and bacteria within the invaginations. Bars, 5 μm or 1 μm (inset). (C’ to C’’’) Line scan analysis of the L. monocytogenes membrane protrusion/invagination from the merge inset in panel C. (C’) Enlargement of the merge inset in panel C. (C’’) A heat map of the representative mDia1 signal from panel C’ (light yellow indicates the highest signal intensity). Bar, 1 μm. (C’’’) A 1.5-μm line (white line) was drawn through the protrusion/invagination (see panel C’). Total F-actin intensity (red) and the corresponding mDia1 intensity (green) were plotted. (D to D’’’) The same as shown in panels D to D’’’ but using HeLa cells in place of Jeg-3 cells. (E to E’’) Mixed HeLa cell assays and quantification of the localization frequency of mDia1-Emerald (mDia1 [E]), p34-Emerald (p34 [E’]) at L. monocytogenes membrane invaginations when expressed in the invagination-forming host cells (green). Endogenous CD147 (red) labels the invaginations in the invagination-forming host cells, while NucBlue (blue) labels bacterial DNA. Solid arrowheads indicate the L. monocytogenes protrusion/invagination, while the open arrowheads indicate the bacteria at the structures. Bars, 5 μm. (E’’) The average percent frequencies of mDia1-Emerald (mDia1-Erld), p34-Emerald (p34-Erld), and the empty Emerald vector (Erld) enrichment at CD147-positive membrane invaginations is presented as a bar graph (mean plus standard deviation [SD] [error bar]). At least 50 CD147-positive bacterial spreading events from at least 18 microscopy field of views were analyzed for each construct tested. The average values are as follows: 85.15% (mDia1-Erld), 4.167% (p34-Erld), and 0% (Erld). ****, *P* < 0.0001 (Kruskal-Wallis test followed by Dunn’s multiple-comparison test). (F and F’) Mixed HeLa cell assays and quantification of the localization frequency of LifeAct-eGFP (LifeAct [F]) at L. monocytogenes membrane invaginations when expressed in the invagination-forming host cells (green). Endogenous CD147 (red) labels the invaginations in the invagination-forming host cells. NucBlue (blue) labels bacterial DNA. Solid arrowheads indicate the L. monocytogenes protrusion/invagination, while the open arrowheads indicate the bacteria at the structures. Bar, 5 μm. (F’) The average percent frequencies of LifeAct-eGFP and the empty eGFP vector (eGFP) enrichment at CD147-positive membrane invaginations is presented as a bar graph (mean ± SD). At least 49 CD147-positive bacterial spreading events from at least 16 microscopy field of views were analyzed for each construct. The average values are as follows: 89.0625% [LifeAct-eGFP] and 0% [eGFP]. ****, *P* < 0.0001 (unpaired Mann-Whitney U test). The white stars in the merge panels indicate the locations of the untransfected protrusion-forming host cells. All insets are enlargements of the boxed regions. Color intensities are enhanced in the insets to clearly visualize the labeled proteins. Solid arrowheads indicate the invagination, and open arrowheads indicate the spreading bacteria.

The absence of the Arp2/3 complex at L. monocytogenes membrane invaginations suggests that the actin shell surrounding the invaginations might be organized as unbranched (linear) actin rather than a branched (dendritic) network. We thus studied the localization of mDia1, a major formin protein (43, 44) that is highly expressed in epithelial cells (44–46), at L. monocytogenes membrane invaginations. We found that endogenous mDia1 accumulated at bacterial cell-to-cell spreading sites in Jeg-3 cells (Fig. 2A). Line scan analysis of endogenous mDia1 at the membrane protrusion/invagination produced the two peaks flanking a single F-actin peak (Fig. 2B to B’’). We used a mixed Jeg-3 cell assay to verify that the host cell forming the bacterial membrane invagination accounted for the observed enrichment of mDia1. Consistent with the above data, we saw elevated levels of mDia1-Emerald delineating the entire length of the invaginations (Fig. 2C). Heat maps confirmed this result, and line scan analysis of mDia1-Emerald at the invaginations generated the characteristic double peak surrounding phalloidin-labeled F-actin (Fig. 2C to C’’’ and Fig. S2A to S2B’’). As expected, mDia1-Emerald also accumulated in a linear-like fashion at the invaginations when we examined membrane invaginations formed in HeLa cells (Fig. 2D to D’’’ and Fig. S2C to S2D’’). Although exogenously expressed mDia1 has been previously identified within L. monocytogenes protrusions (47), we noticed a lack of endogenous mDia1 within the actin-rich core of membrane protrusions engaged in intercellular spreading (Fig. 2B). However, membrane protrusions that extended outwards into extracellular space and were not actively engaged with a neighboring host cell routinely contained elevated levels of mDia1 within their actin-rich cores (Fig. S2E). The enrichment of mDia1 at L. monocytogenes membrane invaginations did not coincide with alterations in the endogenous levels of the protein following 8-h infections of Jeg-3 or HeLa cells compared to uninfected samples (Fig. S2F and S2G).

10.1128/mBio.02939-21.2 FIG S2 Additional characterization of mDia1, p34/Arpc2, and LifeAct at L. monocytogenes membrane invaginations and other bacterially generated structures. (A to D’’) Mixed Jeg-3 (A to B’’) and HeLa (C to D’’) cell assays show mDia1-Emerald (green) concentrated at bacterial membrane invaginations when expressed in solely in the invagination-forming host cells. Samples were stained with Alexa Fluor 594-phalloidin (red) to visualize total F-actin and NucBlue (blue) to visualize host DNA and bacteria within the invaginations. The white stars indicate the locations of the untransfected protrusion-forming host cells. (A’, B’, C’, and D’) Zoomed-in regions from the corresponding boxed images in panels A, B, C and D. Color intensities are enhanced in the zoomed-in images to clearly visualize the localized proteins. Solid arrowheads indicate the invaginations, and open arrowheads indicate spreading bacteria. A 1.5-μm white line was drawn through the area of the invagination/protrusion for pixel intensity profiling. (A”, B”, C”, and D’’) The corresponding pixel intensity plots from the white line in panels A’, B’, C’ and D’. Scale bars are 5 μm and 2 μm (inset). (E) Endogenous mDia1 expectedly localizes within L. monocytogenes membrane protrusions that are protruding into the extracellular space. Jeg-3 cells were infected with L. monocytogenes and stained with mDia1 targeting antibodies (green), NucBlue (blue) to visualize host cell DNA and bacteria, and Alexa Fluor 594-phalloidin (red) to visualize F-actin. Insets shown are an enlargement of the boxed region. Color intensities are enhanced in the insets to clearly visualize the labeled proteins. Solid arrowheads indicate mDia1 enrichment at the bacterial membrane protrusions. Open arrowheads indicate the bacteria within the structures. Scale bars are 5 μm and 2 μm (inset). (F and G) Formin mDia1 protein levels are unaltered during L. monocytogenes infections. Whole Jeg-3 (F) or HeLa (G) cell lysates from uninfected (Uninf) cells versus cells infected with L. monocytogenes for 8 h (Inf [8h]) were probed for endogenous mDia1 using rabbit polyclonal anti-mDia1 (mDia1) antibodies. α-Tubulin is shown as a loading control. Download FIG S2, TIF file, 1.5 MB. Copyright © 2021 Dhanda et al. 2021 Dhanda et al. https://creativecommons.org/licenses/by/4.0/ This content is distributed under the terms of the Creative Commons Attribution 4.0 International license.

To quantify the frequency by which L. monocytogenes membrane invaginations accumulate mDia1, we examined mDia1-Emerald in membrane invagination-forming host cells that were also stained with CD147, a host transmembrane glycoprotein receptor that labels L. monocytogenes membrane invaginations (37). We determined that ∼85% of CD147-positive L. monocytogenes membrane invaginations were delineated with mDia1-Emerald (Fig. 2E and E’’). In contrast, only ∼4% of CD147-positive membrane invaginations contained weakly elevated p34-Emerald levels (Fig. 2E’ to E’’), while the Emerald vector alone failed to localize to the CD147-positive membrane invaginations (Fig. 2E’’). Together, these results support the specificity of mDia1 recruitment to the endocytic sites. Of note, F-actin (LifeAct) also accumulated at a high frequency (∼89%) (Fig. 2F to F’). Collectively, these data point toward mDia1 and F-actin as bona fide host components of L. monocytogenes membrane invaginations.

Next, we assessed the presence and localization of mDia1 at various structures often formed during the bacterial cell-to-cell spreading process. We first examined sites where the bacterium-containing membrane protrusion initially contacts a neighboring host cell membrane and deforms it into a characteristic invagination shape. At these sites (termed “Early”), mDia1-Emerald marked the tip of the invagination, gradually decreasing in intensity distal to the tip (Fig. S3A). The staining also showed the expected double peak in staining intensity across the nascent invagination (Fig. S3A’ to S3A’’). Occasionally, these “early” membrane invaginations contained a single bright punctum of mDia1 at their tips (Fig. S3B to B’’). We then examined membrane invaginations that were deeper in the neighboring host cells. At these invaginations we often observed a thin trailing streak (a tether) at the distal end of the invaginations that presumably resulted from the close interaction of the plasma membrane as it wrapped around the internalized bacterium prior to its scission and release of the bacterium-containing double-membrane vacuole. mDia1-Emerald and F-actin routinely concentrated at these tethers (Fig. S3C to S3E). This is in line with a recent study that showed caveolin-1 also present at the tether-like structures (26). Finally, we examined encircled L. monocytogenes that were presumably generated following a scission event. Those structures were positive for both mDia1 and F-actin (Fig. S3F to S3G’’).

10.1128/mBio.02939-21.3 FIG S3 Characterization of mDia1 and LifeAct at L. monocytogenes membrane invaginations during various stages of their development in the invagination-forming cell. (A to G’’) Mixed HeLa cell assays demonstrating mDia1-Emerald (green) (or LifeAct-eGFP [green]) localization at various stages of membrane invagination formation. Fixed samples were stained with Alexa Fluor 594-phalloidin (red) to visualize actin (or CD147-targeting antibodies [red] to visualize vacuole-like structures) and with NucBlue (blue) to confirm the presence of bacteria at the structures. White stars indicate the locations of the untransfected protrusion-forming host cells. Solid arrowheads indicate mDia1 (or LifeAct) localization, and open arrowheads indicate the locations of the spreading bacteria. (A’, B’, C’, D’, F’, and G’) Heat maps of the representative mDia1 (or LifeAct) signal (light yellow indicates the highest signal intensity). (A’’, B’’, C’’, D’’, F’’, and G’’) A 1.5- to 2.5-μm white line was drawn through the corresponding structures (see panels A, B, C, D, F, and G), and the total F-actin intensity (red) as well as the corresponding mDia1 (or LifeAct) intensity (green) was plotted. (A, “Early’) Initial contact of a membrane protrusion (from an untransfected host cell) with a neighboring mDia1-Emerald-expressing host cell. (B, “Tip”) Initial contact of a membrane protrusion (from an untransfected host cell) with a neighboring mDia1-Emerald-expressing host cell showing a punctum of mDia1 at the invagination tip. (C and D, “Tether”) Deep invaginations sealed at their distal ends with a trailing membrane strand (or tether) found within the mDia1-Emerald-expressing (C) or LifeAct-eGFP-expressing (D) host cell. (E) An expanded image from panel D depicting LifeAct-eGFP (expressed in the invagination-forming host cell) localizing at the trailing membrane strands (or tethers) of multiple membrane invaginations. Color intensities are enhanced in the zoomed-in images (enlargement of the color-coded boxes) to clearly visualize the LifeAct signal. Scale bars are 5 μm and 1 μm (insets). (F and G, “Sealed”) Vacuole-like structures formed from resolved invaginations found within the mDia1-Emerald-expressing (F) or Life-eGFP-expressing (G) host cell. Scale bars are 1 μm except in panels C to D’, which are 2 μm. Download FIG S3, TIF file, 1.7 MB. Copyright © 2021 Dhanda et al. 2021 Dhanda et al. https://creativecommons.org/licenses/by/4.0/ This content is distributed under the terms of the Creative Commons Attribution 4.0 International license.

### An mDia1-dependent linear F-actin shell coats L. monocytogenes membrane invaginations.

**FIG 3:**
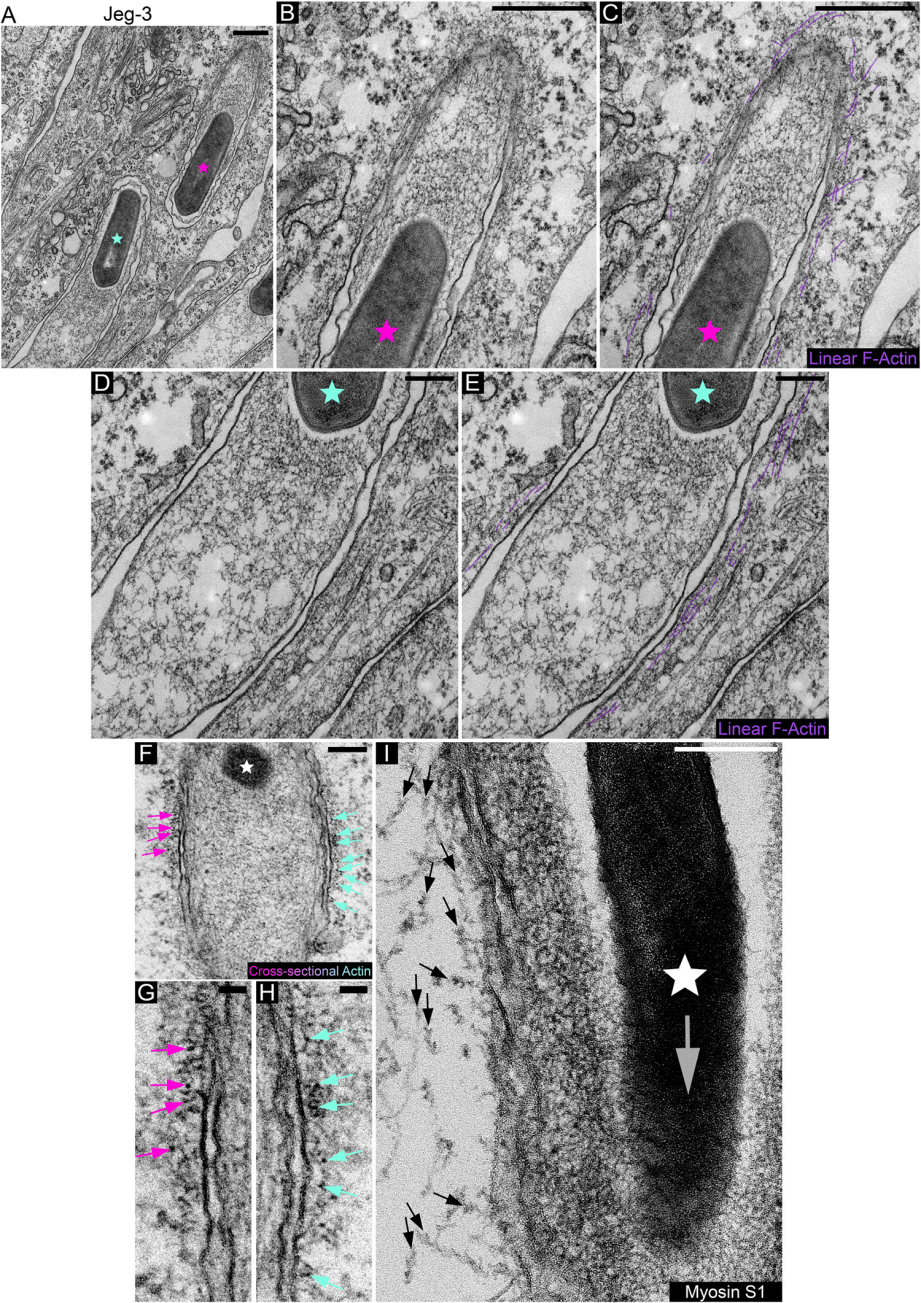
Electron microscopic examination and myosin S1 decoration of linear actin filaments surrounding L. monocytogenes membrane invaginations. (A to E) Linear actin filaments surround L. monocytogenes membrane invaginations formed in Jeg-3 cells. (B to E) Enlargement of the membrane invaginations depicted in panel A. The pink and light blue stars correspond to the locations of the bacteria in panel A. Linear F-actin filaments are highlighted in purple in panels C and E. Bars, 500 nm (A to C) and 200 nm (D and E). (F) Cross-sectional actin filaments run perpendicular to the plane of the L. monocytogenes membrane invagination. The white star corresponds to the location of the bacterium. Pink and blue arrows point to cross-sectional actin filaments (depicted as electron-dense puncta). (G and H) Enlargements of panel F depicting the same color coded arrows. Bars, 200 nm (F) and 50 nm (G and H). (I) Myosin S1 decoration of actin filaments surrounding L. monocytogenes membrane invaginations formed in Jeg-3 cells. Arrows indicate myosin S1-decorated linear actin strands as well as point in the direction of the filament pointed ends. The white star corresponds to the location of the bacterium. The large translucent/gray arrow points in the direction of bacterial spread into the receiving invagination-forming host cell. Bar, 200 nm.

The presence of mDia1 at L. monocytogenes membrane invaginations raises the possibility that the actin surrounding the structures is organized into linear arrays. To examine this, we used transmission electron microscopy. We found that linear actin filaments lined the cytoplasmic area of the invaginations, appeared short in length, and were often arranged in parallel fashion while also forming a layer not exceeding three filaments in thickness (Fig. 3A to E). Next, we made use of myosin subfragment 1 (S1) to examine the orientation of the actin filaments. When cross sections of the invaginations were examined in glycerol-extracted and tannic acid-treated cells, in which the myosin S1 had not reached the filaments, the cytoplasmic side of the invagination membranes showed electron-dense clusters studded along the plane of the invagination membrane in what appeared to be close-packed formations, which are characteristic of parallel actin (Fig. 3F to H). In cells where the actin filaments were decorated with S1, we found that the barbed ends of the linear actin arrays surrounding the bacterial membrane invagination were oriented opposite to the direction of bacterial spread (Fig. 3I). This is in agreement with findings in a recent study showing F-actin accumulating toward the basal region of the protrusion/plasma membrane of the invagination-forming host cell (38). Given that some formins are able to both nucleate and elongate linear actin filaments (43, 44) and because mDia1 is expressed at high levels in many epithelial cell lines (45, 46), the observed enrichment of mDia1 within the actin shell suggests that its recruitment could be either a prerequisite for its assembly or a consequence of it.

**FIG 4:**
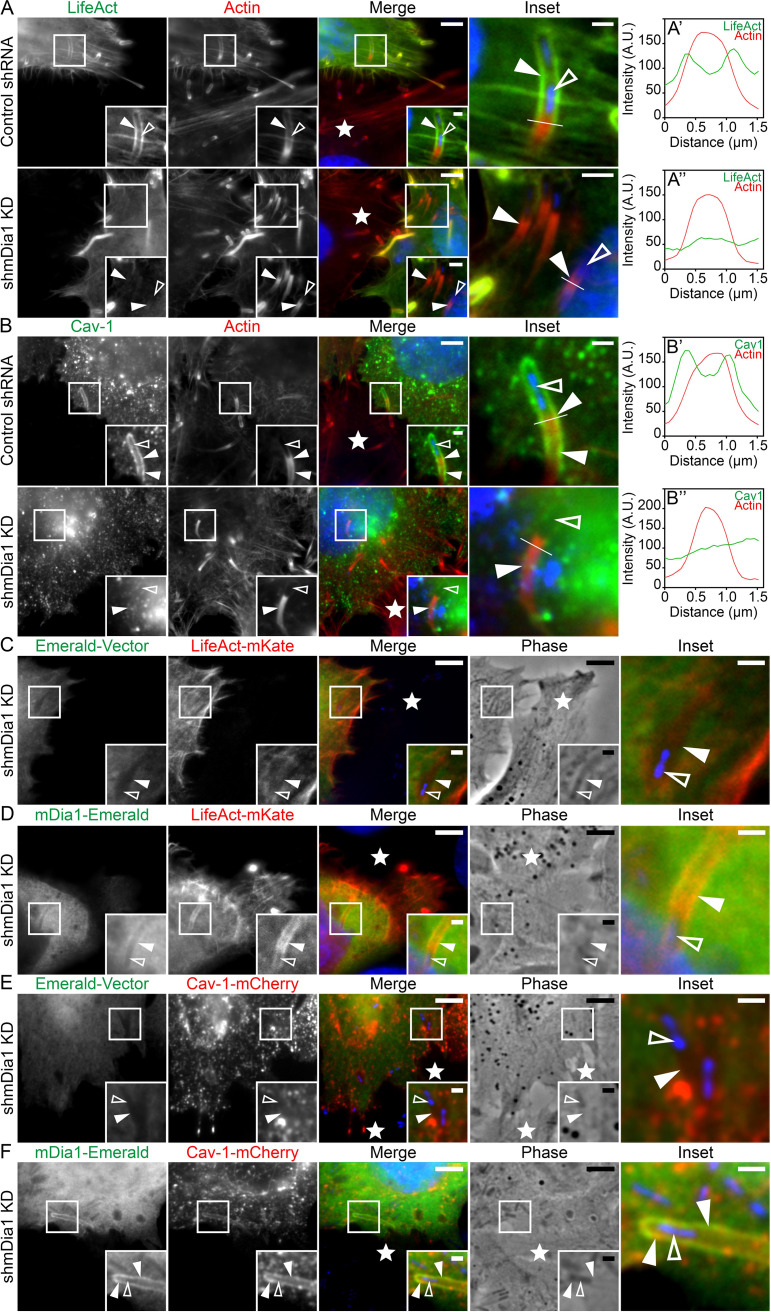
mDia1 expression is crucial for actin formation and maintaining caveolin-1 at L. monocytogenes membrane invaginations. (A to A’’) mDia1 expression is crucial for F-actin polymerization around L. monocytogenes membrane invaginations. HeLa cells transfected with LifeAct-eGFP (green) and stably expressing control [nontargeting] shRNA (top row, Control shRNA) or mDia1-targeting shRNA (bottom row, shmDia1 knockdown [KD]) were overlaid with unlabeled preinfected (protrusion-forming) HeLa (Control shRNA) cells. Samples were stained with Alexa Fluor 594-phalloidin (labeled “Actin”; red) to visualize total F-actin and NucBlue (blue) to label bacteria within the membrane invaginations. The solid arrowheads indicate the presence (top row) or absence (bottom row) of mDia1 at the bacterial membrane invaginations. Bars = 5 μm or 1 μm (insets) (top row) and 5 μm or 2 μm (insets) (bottom row). (A’ to A’’) Line scan analysis of the L. monocytogenes membrane protrusion/invagination from the corresponding merge insets in panel A. A 1.5-μm white line was drawn through the protrusion/invagination (see the insets in panel A) and the total F-actin intensity (red) as well as the corresponding LifeAct intensity (green) was plotted. (B) Formin mDia1 expression is crucial for high-level caveolin-1 localization around L. monocytogenes membrane invaginations. HeLa cells transfected with caveolin-1-mCherry (pseudo-colored green) and stably expressing control [nontargeting] shRNA (top row, Control shRNA) or mDia1-targeting shRNA (bottom row, shmDia1 KD) were overlaid with unlabeled preinfected (protrusion-forming) HeLa (Control shRNA) cells. Samples were stained with Alexa Fluor 488-phalloidin (labeled “Actin”; pseudo-colored red) to visualize F-actin and NucBlue (blue) to confirm the presence of bacteria within the membrane invaginations. Solid arrowheads indicate caveolin-1-mCherry presence (top row) or depletion (bottom row) at the bacterial membrane invaginations. Open arrowheads indicate the location of the spreading bacteria. Bars, 5 μm or 1 μm (insets). (B’ and B’’) Line scan analysis of the L. monocytogenes membrane protrusion/invagination from the corresponding merge insets in panel B. A 1.5-μm white line was drawn through the protrusion/invagination (see the “Inset” panels in panel B). Total F-actin intensity (red) and the corresponding caveolin-1 intensity (green) were plotted. (C and D) Transfection of mDia1-depleted cells (shmDia1 KD) with mDia1-Emerald restores F-actin polymerization around bacterial membrane invaginations. Formin mDia1-depleted HeLa cells (shmDia1 KD) were cotransfected with LifeAct-mKate (red) and the empty Emerald vector (green [C]) or LifeAct-mKate (red) and mDia1-Emerald (green [D]). These cells were overlaid with unlabeled preinfected (protrusion-forming) HeLa (Control shRNA) cells. Fixed samples were stained with NucBlue (blue) to confirm the presence of bacteria within the membrane invaginations. The solid arrowheads indicate LifeAct absence (C) or presence (D) at the bacterial membrane invaginations. The open arrowheads indicate the locations of the spreading bacteria. Bars, 5 μm or 1 μm (insets). (E and F) Transfection of mDia1-depleted cells (shmDia1 KD) with mDia1-Emerald restores caveolin-1 at bacterial membrane invaginations. mDia1-depleted HeLa cells (shmDia1 KD) were cotransfected with caveolin-1-mCherry (red) and the empty Emerald vector (green [E]) or caveolin-1-mCherry (red) and mDia1-Emerald (green [F]). These cells were overlaid with unlabeled preinfected (protrusion-forming) HeLa (Control shRNA) cells. Fixed samples were stained with NucBlue (blue) to confirm the presence of bacteria within the membrane invaginations. The solid arrowheads indicate the absence (E) or presence (F) of caveolin-1 at the bacterial membrane invaginations. The open arrowheads indicate the locations of the spreading bacteria. Bars, 5 μm or 1 μm (insets). The white stars in the merge panels indicate the locations of the untransfected protrusion-forming host cells. All insets are enlargements of the boxed regions. Color intensities are enhanced in the insets to clearly visualize the labeled proteins. Open arrowheads indicate the spreading bacteria.

To investigate this issue, we took advantage of a well-characterized stable mDia1 knockdown HeLa cell line (45). Western blotting confirmed that the mDia1 protein levels were reduced by ∼90% in the mDia1-depleted cells compared to control cells (Fig. S4A and B). Upon infection, cytosolic L. monocytogenes could still generate actin clouds, comet/rocket tails, and membrane protrusions in the mDia1 knockdown cells (data not shown). These results confirm a previous study in which the pan-formin inhibitor SMIFH2 or the small interfering RNA (siRNA)-mediated depletion of mDia formins were employed (47). However, because L. monocytogenes membrane protrusions are reported as being ∼25% shorter in mDia1-depleted cells (47), these defective protrusions might influence the morphology of the membrane invaginations. Thus, to assess the role of mDia1 at the bacterial membrane invaginations, we utilized mixed-cell assays by preinfecting control short hairpin RNA (shRNA) cells with L. monocytogenes and then mixing the infected cells with uninfected mDia1 knockdown cells. Bacterial cell-to-cell spreading events revealed that the bacterial membrane protrusions could still induce the formation of membrane invaginations in mDia1-depleted cells. Yet, these membrane invaginations were no longer surrounded by F-actin (Fig. 4A) as also shown by line scan analysis of the LifeAct signal across the membrane invaginations (Fig. 4A’ to A’’). Interestingly, we also observed a dramatic reduction in caveolin-1 levels at the invaginations that formed in the mDia1-depleted cells (Fig. 4B to B’’). Importantly, this was not due to a reduction in whole-cell caveolin-1 protein levels (Fig. S4C).

10.1128/mBio.02939-21.4 FIG S4 Additional characterization of stable mDia1 knockdown HeLa cells and examination of mDia1 and LifeAct at membrane invaginations generated in p34/ArpC2-depleted cells. (A) HeLa cells stably transfected with mDia1-targeting shRNA (mDia1) or nontargeting control shRNA (Control). Whole-cell lysates from the cells were collected and probed for endogenous mDia1 using rabbit polyclonal mDia1-targeting antibodies. α-Tubulin is shown as a loading control. (B) Quantification of mDia-1 protein levels from panel A. Six lanes each from samples of nontargeting control shRNA [Control]- and mDia1 shRNA [mDia1]-expressing cells were analyzed. Percent values (relative to control [± standard deviation {s.d.}]) are 100% [Control shRNA] and 11.16% [mDia1 shRNA]. ***, *P* < 0.001 (unpaired Mann-Whitney U test). (C) Whole-cell lysates of cells stably transfected with nontargeting control shRNA [Control] or mDia1-targeting shRNA [mDia1] were collected and probed for endogenous caveolin-1. α-Tubulin is shown as a loading control. (D and E) Expression of the Arp2/3 complex in invagination-forming host cells is dispensable for F-actin (D) and mDia1 (E) localization at L. monocytogenes membrane invaginations. Control wild-type and p34/AprC2 knockout (*ArpC2^−/−^*) MEFs transfected with LifeAct-eGFP (green) (D) or mDia1-Emerald (green) (E) were overlaid with unlabeled preinfected (protrusion-forming) control wild-type cells. Samples were stained with Alexa Fluor 594-phalloidin (red) to visualize F-actin and NucBlue (blue) to confirm the presence of bacteria within the membrane invaginations. The white stars indicate the locations of the untransfected protrusion-forming host cells. Insets are enlargements of the boxed regions. Enlargements of the merge inset are depicted in the fourth column. Color intensities are enhanced in the insets (and enlarged merge insets) to more clearly visualize the labeled proteins. Solid arrowheads indicate protein localization at the bacterial membrane invaginations. Open arrowheads indicate the locations of the spreading bacteria. Scale bars are 5 μm or 1 μm (insets). Download FIG S4, TIF file, 1.4 MB. Copyright © 2021 Dhanda et al. 2021 Dhanda et al. https://creativecommons.org/licenses/by/4.0/ This content is distributed under the terms of the Creative Commons Attribution 4.0 International license.

To confirm that the F-actin coat lining the membrane invaginations assembles independently of the Arp2/3 complex, we took advantage of the fact that the ablation of the *Arpc2* gene (encoding ARPC2/p34) results in the downregulation of several other Arp2/3 complex subunits, including Arp2 and Arp3 (48). As L. monocytogenes is unable to generate comet/rocket tails in an established line of *Arpc2^−/−^* fibroblasts (48, 49), we preinfected wild-type cells with L. monocytogenes and then overlaid these cells onto culture dishes containing either uninfected wild-type cells or *Arpc2^−/−^* cells transfected with mDia1-Emerald or fluorescent LifeAct. These experiments revealed the presence of both mDia1 and F-actin (LifeAct) at the membrane invaginations generated in either wild-type or Arp2/3 knockout host cells (Fig. S4D and E).

More importantly, the reexpression of mDia1 in the mDia1 knockdown cells could rescue the F-actin coat at L. monocytogenes membrane invaginations. The presence of F-actin at the invaginations was evident in the mDia1 knockdown cells cotransfected with mDia1-Emerald and LifeAct-mKate (Fig. 4D), but not in those expressing the Emerald vector and LifeAct-mKate (Fig. 4C). Rescue of caveolin-1 localization at membrane invaginations also occurred in mDia1 knockdown cells transfected with mDia1-Emerald and caveolin-1-mCherry (Fig. 4E and F).

The sum of these data indicates that an mDia1-dependent linear F-actin coat surrounds L. monocytogenes membrane invaginations and that this influences the presence of caveolin-1 at these endocytic structures.

### The linear F-actin coat of L. monocytogenes membrane invaginations contains caveola-associated F-actin-binding proteins.

The dependence of caveolin-1 localization on mDia1 and F-actin as well as the regular spacing of the linear actin filaments that make up the coat at bacterial membrane invaginations prompted us to look at Filamin A and myosin 1c, which are established caveola-associated F-actin-binding proteins (50–54; see reference 55 for a review).

**FIG 5:**
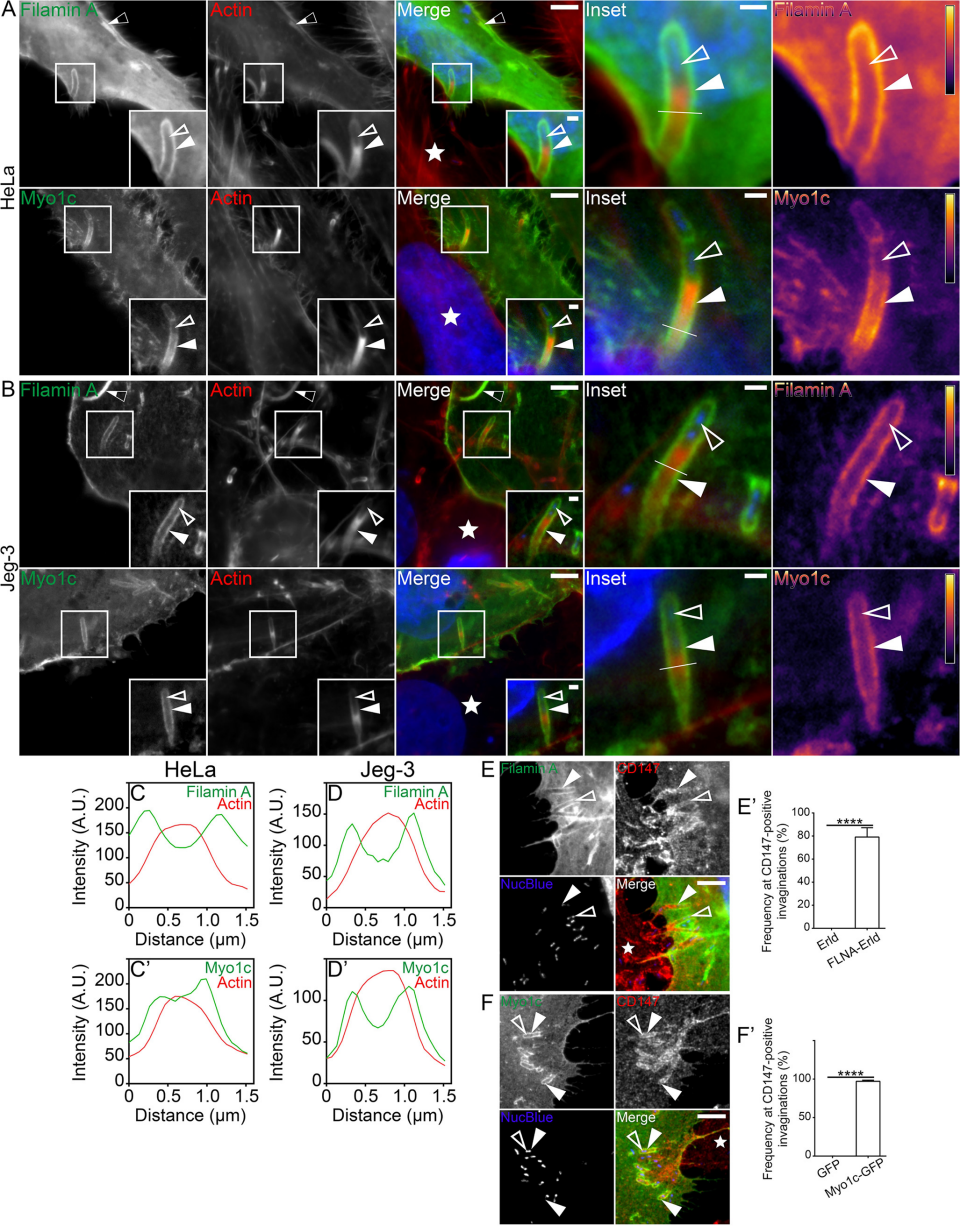
Caveola-associated F-actin-binding proteins are enriched at L. monocytogenes membrane invaginations. (A and B) Mixed HeLa (A) and Jeg-3 (B) cell assays show Filamin A-Emerald (green) and Myo1c-GFP (green) concentrated at L. monocytogenes membrane invaginations when expressed in the invagination-forming host cells. Samples stained with Alexa Fluor 594-phalloidin (red) to visualize F-actin and NucBlue (blue) to visualize host DNA and bacteria within the invaginations. Heat maps (rightmost column) of the representative Filamin A or Myo1c signal based on the insets (light yellow indicates the highest signal intensity). Bars, 5 μm or 1 μm (inset). (C to D’) Line scan analyses of the L. monocytogenes membrane protrusion/invagination from the enlarged merge insets found in panels A and B. A 1.5-μm line (white line) was drawn through the protrusion/invagination (see the enlarged merge insets found in panels A and B). Total F-actin intensity (red) as well as the corresponding Filamin A or Myo1c intensity (green) was plotted. (E and E’) Mixed HeLa cell assays and quantification of the localization frequency of Filamin A-Emerald (Filamin A) at L. monocytogenes membrane invaginations when expressed in the invagination-forming host cells (green). Endogenous CD147 (red) labels the invaginations in the invagination-forming host cells. NucBlue (blue) labels bacterial DNA. Solid arrowheads indicate the L. monocytogenes protrusion/invagination. Open arrowheads indicate the bacteria at the structures. The white star indicates the location of the untransfected protrusion-forming host cells. Bar, 5 μm. (E’) The average percent frequencies of Filamin A-Emerald (FLA-Erld) and the empty Emerald vector (Erld) enrichment at CD147-positive membrane invaginations are presented as a bar graph (mean plus SD). At least 47 CD147-positive bacterial spreading events from 20 microscopy field of views were analyzed for each construct tested. The average values are as follows: 79.17% (FLNA-Erld) and 0% (Erld). ****, *P* < 0.0001 (unpaired Mann-Whitney U test). (F and F’) Same as in panels A and A’ but examining Myo1c-GFP in place of Filamin A-Emerald. The average frequencies of Myo1c-GFP and the empty GFP vector (GFP) enrichment at CD147-positive membrane invaginations are presented as a bar graph (mean plus SD). At least 83 CD147-positive bacterial spreading events from 18 microscopy field of views were analyzed for each construct tested. The average values are as follows: 97.15% (Myo1c-GFP) and 0% (GFP). ****, *P* < 0.0001 (unpaired Mann-Whitney U test). The white star indicates the location of the untransfected protrusion-forming host cell. Insets are enlargements of the boxed regions. Color intensities are enhanced in the insets (and enlarged merge insets) to clearly visualize the labeled proteins. Solid arrowheads indicate the membrane invaginations, and open arrowheads indicate the spreading bacteria. Half-filled arrows depict Filamin A localizing at cytoplasmic comet/rocket tails.

Using our mixed HeLa cell assay, we found that both Filamin A-Emerald and Myo1c-GFP were enriched along the entire length of the L. monocytogenes membrane invaginations (Fig. 5A). These observations were confirmed in Jeg-3 cells (Fig. 5B). Line scan analysis of Filamin A-Emerald and Myo1c-GFP at the structures also generated the characteristic double peak surrounding actin (Fig. 5C and D’). Filamin A-Emerald and Myo1c-GFP localization also remained throughout the length of invaginations that were connected to the cell edge via thin membranous tethers (Fig. S5A to S5B’’). In good agreement with the localization of mDia1 and F-actin at the structures, ∼79% and ∼97% of the CD147-positive membrane invaginations on average were also positive for Filamin A and Myo1c, respectively (Fig. 5E and F’). Strikingly, the presence of Filamin A, but not Myo1c, at L. monocytogenes membrane invaginations heavily relied on mDia1 and, by extension, the presence of the F-actin coat (Fig. S6A to S6F’’). These observations suggest that Filamin A recruitment is actin dependent, whereas that of Myo1c is not. As expected, both actin-binding proteins were recruited to L. monocytogenes membrane invaginations generated in *Arpc2^−/−^* cells (Fig. S6G to L’’). Thus, although two caveola-associated actin-binding proteins decorate L. monocytogenes membrane invaginations, only the recruitment of Filamin A to these noncanonical caveolin-rich endocytic structures relies on an mDia1-assembled F-actin coat.

10.1128/mBio.02939-21.5 FIG S5 Characterization of Filamin A and Myo1c at L. monocytogenes membrane invaginations and other bacterially induced actin-rich structures. (A to B’’) Mixed HeLa cell assays demonstrating Filamin A-Emerald (A to A’’) or Myo1c-GFP (B to B’’) (green) localization at invaginations sealed at their distal ends with a trailing membrane strand (or tether). Samples were stained with Alexa Fluor 594-phalloidin (red) to visualize actin and with NucBlue (blue) to confirm the presence of bacteria at the structures. Scale bars are 2 μm. (A’ to B’) Heat maps of the representative Filamin A (A’) or Myo1c (B’) signal (light yellow indicates the highest signal intensity). (A’’ to B’’) A 1.5-μm line (white line) was drawn through the corresponding structures (see panels A and B). Total F-actin intensity (red) as well as the corresponding Filamin A (A’’) or Myo1c (B’’) intensity (green) was plotted. (C to G) HeLa cells were transfected with Filamin A-Emerald or Myo1c-GFP (green) and then infected with L. monocytogenes to examine their localization at other bacterial actin-rich structures. Samples stained with Alexa Fluor 594-phalloidin (red) to visualize F-actin (or CD147-targeting antibodies to label membrane protrusions [red] [D and E]) and NucBlue (blue) to visualize host DNA and bacteria. Solid arrowheads indicate Filamin A (or Myo1c) localization (or absence). Open arrowheads indicate the bacteria at the corresponding actin-rich structures. Insets are enlargements of the boxed regions. Color intensities are enhanced in the insets to more clearly visualize the labeled proteins. (C) Filamin A localizes to cytoplasmic comet/rocket tails as well as actin clouds (half-filled arrowheads indicate the actin cloud of interest and also correspond to the small insets at the bottom left corner of each panel). (D) Filamin A localizes to CD147-positive membrane protrusions. (E) Myo1c localizes to CD147-positive membrane protrusions. The half-filled arrowheads indicate an additional Myo1c- and CD147-positive membrane protrusion. (F) Myo1c does not localize to cytoplasmic comet/rocket tails or actin clouds (half-filled arrowheads indicate the actin clouds of interest and also correspond to the small insets at the bottom left corner of each panel). (G) Myo1c-GFP signal at L. monocytogenes spreading events is intensified when Myo1c-GFP is expressed in both the protrusion-forming and invagination-forming cells. Scale bars are 10 μm, 2 μm (large insets), and 1 μm (small insets [C and F]). Download FIG S5, TIF file, 2.6 MB. Copyright © 2021 Dhanda et al. 2021 Dhanda et al. https://creativecommons.org/licenses/by/4.0/ This content is distributed under the terms of the Creative Commons Attribution 4.0 International license.

10.1128/mBio.02939-21.6 FIG S6 Characterization of Filamin A and Myo1c at membrane invaginations generated in mDia-1-depleted or p34/ArpC2-depleted cells. (A to C’’) Formin mDia1 expression is crucial for Filamin A localization around L. monocytogenes membrane invaginations. HeLa cells transfected with Filamin A-Emerald (green) and stably expressing control [nontargeting] shRNA (top row in panel A, Control shRNA) or mDia1-targeting shRNA (bottom row in panel A, shmDia1 KD) were overlaid with unlabeled preinfected (protrusion-forming) HeLa (Control shRNA) cells. Samples were stained with Alexa Fluor 594-phalloidin (red) to visualize F-actin and NucBlue (blue) to confirm the presence of bacteria within the membrane invaginations. The white stars indicate the locations of the untransfected protrusion-forming host cells. Scale bar is 5 μm. (B and C) The color-coded insets are enlargements of the same color-coded boxed regions in panel A. Color intensities are enhanced in the insets to more clearly visualize the labeled proteins. The solid arrowheads indicate Filamin A presence (B) or absence (C) at the bacterial membrane invaginations. The open arrowheads indicate the locations of the spreading bacteria. Scale bars are 1 μm. (B’ and C’) Heat maps of the representative Filamin A signal (light yellow indicates the highest signal intensity). (B’’ and C’’) Line scan analyses of the L. monocytogenes membrane protrusion/invagination from the corresponding insets in panels B and C, respectively. A 1.5-μm line (white line) was drawn through the corresponding structures (see panels B and C) and the total F-actin intensity (red) as well as the corresponding Filamin A intensity (green) was plotted. (D to F’’) The same as in panels A to C’’ except examining Myo1c (Myo1c-GFP) in place of Filamin A-Emerald. Expression of mDia1 is dispensable for Myo1c localization at L. monocytogenes membrane invaginations. (G to L’’) Expression of the Arp2/3 complex in invagination-forming cells is dispensable for Filamin A (G to I’’) and Myo1c (J to L’’) localization at L. monocytogenes membrane invaginations. Control wild-type) and p34/AprC2 knockout (*ArpC2^−/−^*) MEFs transiently transfected with Filamin A-Emerald(green) (G to I’’) or Myo1c-GFP (green) (J to L’’) were overlaid with unlabeled preinfected (protrusion-forming) control wild-type cells. Samples were stained with Alexa Fluor 594-phalloidin (red) to visualize F-actin and NucBlue (blue) to confirm the presence of bacteria within the membrane invaginations. The white stars indicate the location sof the untransfected protrusion-forming host cells. Scale bar is 5 μm. (H, I, K, and L) The color-coded insets are enlargements of the same color-coded boxed regions in panels G and J. Color intensities are enhanced in the insets to more clearly visualize the labeled proteins. Solid arrowheads indicate protein localization at the bacterial membrane invaginations. Open arrowheads indicate the location sof the spreading bacteria. Scale bar is 1 μm. (H’, I’, K’, and L’) Heat maps of the representative Filamin A (or Myo1c) signal (light yellow indicates the highest signal intensity). (H’’, I’’, K’’, and L’’) Line scan analyses of the L. monocytogenes membrane protrusion/invagination from the corresponding insets in panels H, I, K, and L, respectively. A 1.5-μm line (white line) was drawn through the corresponding structures (see panels H, I, K, and L). Total F-actin intensity (red) as well as the corresponding Filamin A (or Myo1c) intensity (green) were plotted. Download FIG S6, TIF file, 2.6 MB. Copyright © 2021 Dhanda et al. 2021 Dhanda et al. https://creativecommons.org/licenses/by/4.0/ This content is distributed under the terms of the Creative Commons Attribution 4.0 International license.

Consistent with these conclusions, Filamin A-Emerald also decorated the actin clouds surrounding immotile bacteria (Fig. S5C) and the comet/rocket tails necessary for L. monocytogenes intracellular motility (Fig. S5C), the latter as previously reported (56). Moreover, Filamin A-Emerald was also enriched within the bacterial membrane protrusions (Fig. S5D). In line with the reported localization of Myo1c to the plasma membrane and actin-rich membrane structures (53, 54, 57–62), we observed elevated levels of Myo1c-GFP at the plasma membrane surrounding L. monocytogenes membrane protrusions (Fig. S5E). However, Myo1c-GFP did not accumulate at cytoplasmic actin clouds or comet/rocket tails (Fig. S5F). Interestingly, there was an obvious increase in Myo1c-GFP signal at bacterial cell-to-cell spreading sites engaging two Myo1c-GFP-expressing cells (Fig. S5G). The presence of Myo1c-GFP at both the membrane protrusion and membrane invagination most likely accounts for the observed increase in signal intensity.

### Optimal L. monocytogenes cell-to-cell spread relies on mDia1 expression in the membrane protrusion-receiving cells.

**FIG 6:**
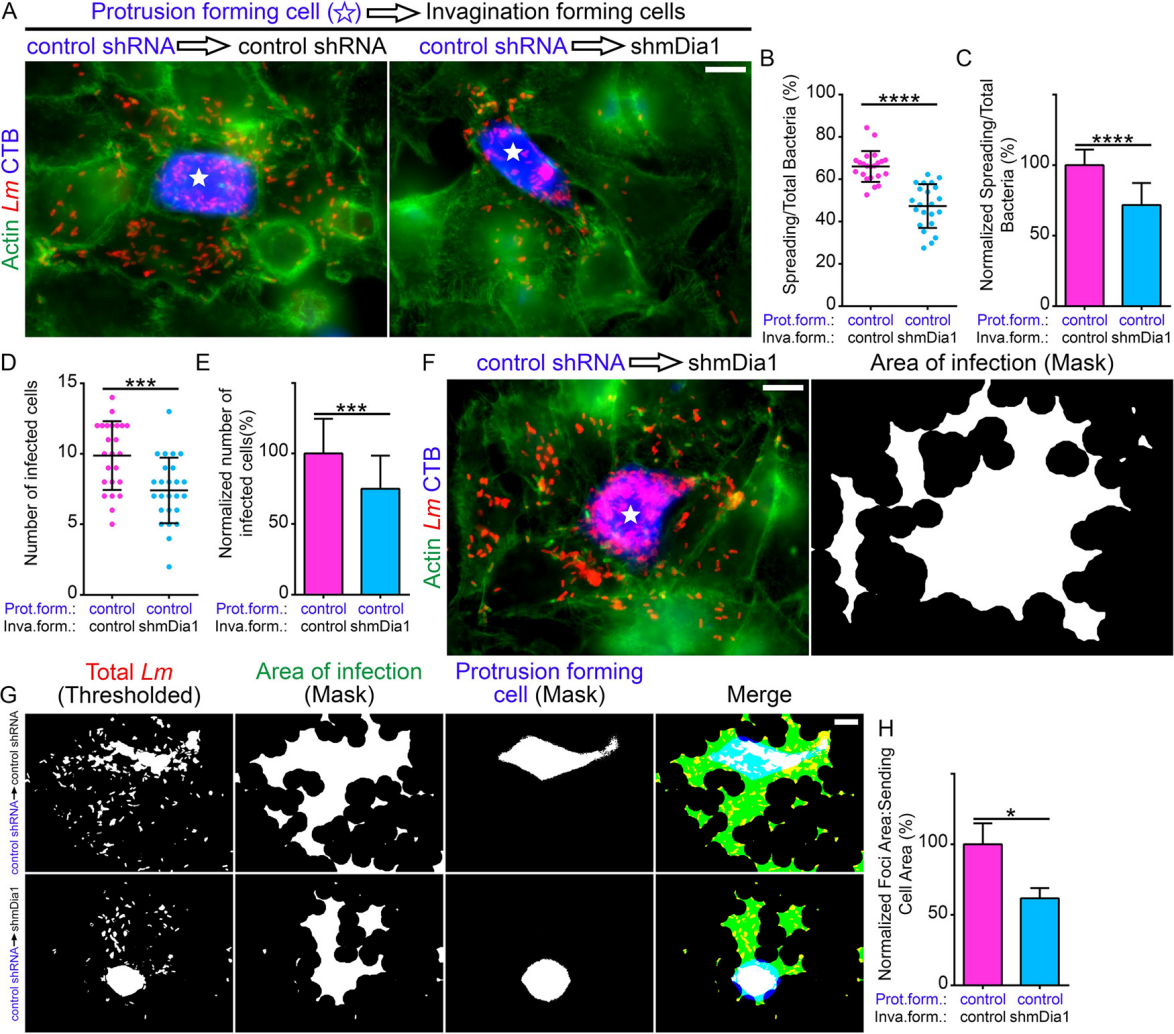
Optimal L. monocytogenes cell-to-cell spread relies on mDia1 expression in the bacterial membrane invagination-forming cells. (A) Micrographs representative of the mixed-cell spreading assays. Preinfected HeLa cells stably expressing the control (nontargeting) shRNA (control shRNA) and labeled with CellTracker Blue (CTB) (blue) were mixed with uninfected (and unlabeled) control (control shRNA) or mDia1-depleted (shmDia1) cells. Samples were stained with anti-L. monocytogenes antibodies (*Lm*) (red) and Alexa Fluor 488-phalloidin (“Actin”) (green) to visualize F-actin. Bar, 10 μm. The white stars indicate the locations of the protrusion-forming host cells. (B) Quantification of the proportion of bacteria that spread (into surrounding nonblue invagination-forming [termed “inva.form.” on the graphs] host cells) out of the CellTracker Blue-labeled protrusion-forming [“prot.form.” on the graphs] host cells. Twenty-two infection foci containing a total of 6,961 bacteria from samples containing control shRNA invagination-forming host cells (pink circles) were analyzed. The same number of infection foci (with a total of 7,846 bacteria) containing mDia1-depleted (shmDia1) invagination-forming host cells (blue circles) were also analyzed. The average ratios (expressed as a percent) of spreading bacteria to total bacteria (depicted as a scatterplot [mean ± SD]) are 66.01% (foci with control shRNA invagination-forming cells) and 47.33% (foci with shmDia1 invagination-forming cells). ****, *P* < 0.0001 (unpaired parametric two-tailed *t* tests [with Welch’s correction]). (C) The data from panel B but normalized to the control (foci with control shRNA invagination-forming cells). Percent values (relative to control, [mean plus SD]) are 100% (foci with control shRNA invagination-forming cells [pink]) and 71.6979% (foci with shmDia1 invagination-forming cells [blue]). ****, *P* < 0.0001 (unpaired parametric two-tailed *t* tests [with Welch’s correction]). (D) Quantification of the number of infected non-CellTracker Blue-labeled cells from the mixed-cell spreading assays. A total of 24 and 25 infection foci containing the control shRNA or shmDia1 invagination-forming cells, respectively, were analyzed. The average number of infected cells (depicted as a scatterplot [mean ± SD]) are 9.875 (foci with control shRNA invagination-forming cells [pink]) and 7.4 (foci with shmDia1 invagination-forming cells [blue]). ***, *P* < 0.001 (unpaired parametric two-tailed *t* tests [with Welch’s correction]). (E) The data from panel D but normalized to the control (foci with control shRNA invagination-forming cells). Percent values (relative to control [mean plus SD]) are 100% (foci with control shRNA invagination-forming cells [pink]) and 74.94% (foci with shmDia1 invagination-forming cells [blue]). ***, *P* < 0.001 (unpaired parametric two-tailed *t* tests [with Welch’s correction]). (F) An example of an infection focus from a mixed-cell spreading assay where the L. monocytogenes bacteria (red) are highly concentrated in the CellTracker Blue-labeled (blue) infected control (control shRNA) protrusion-forming host cell. Bar, 10 μm. The right image depicts the area of infection based on a mask of the thresholded bacteria. (G and H) Quantification of the area of infection by determining the ratio of the total area of infection (green) to the area of the CellTracker Blue-labeled protrusion-forming host cell (“Protrusion Forming Cell”; blue). A mask of the area of infection was created by thresholding the signal of the antibody-labeled L. monocytogenes (red). (H) The average ratios (normalized and expressed as a percentage) of the infection foci area to sending cell area (depicted as a bar graph [mean plus SD]) are 100% (foci with control shRNA invagination-forming cells [pink]) and 61.7289% (foci with shmDia1 invagination-forming cells [blue]). *, *P* < 0.05 (*P* is 0.241 by unpaired Mann-Whitney U test). A total of 10 microscopic field of views and 11 microscopic field of views were analyzed from samples containing control shRNA or shmDia1 invagination-forming cells, respectively.

We next wanted to determine the importance of mDia1 and by extension F-actin accumulation at L. monocytogenes membrane invaginations during bacterial intercellular spreading. To test this, we utilized mixed-population cell-to-cell spreading assays (25, 63) in which CellTracker Blue-labeled control HeLa cells preinfected with L. monocytogenes were mixed with unlabeled (and uninfected) control or mDia1 knockdown HeLa cells. In this way, we quantified the number of bacteria that spread out of an individual infected CellTracker Blue-labeled cell directly into the surrounding unlabeled cells. We determined that, on average, 66% of bacteria spread out of the blue-labeled protrusion-forming cells, when these cells were surrounded by control invagination-forming cells (Fig. 6A and B). Interestingly, bacterial spreading dropped to ∼47% when the surrounding invagination-forming cells were depleted of mDia1 (Fig. 6A and B). Normalized, this corresponds to a significant 30% reduction in bacterial spreading (Fig. 6C). We also enumerated the average number of non-CellTracker Blue-labeled infected cells per foci: ∼10 cells were infected per focus in samples containing control invagination-forming cells, whereas only ∼7 infected cells per focus were observed when the surrounding host cells were depleted of mDia1 (Fig. 6D), hence a 25% reduction (Fig. 6E). Of note, we also saw several infection foci that contained an innumerable number of bacteria packed within the protrusion-forming host cell (Fig. 6F). We observed a similar phenotype when the infection foci contained caveolin-1- or epsin-1-depleted cells (26).

Next, we performed a detailed morphometric analysis of the bacterial infection foci. We determined the ratio of the infected area to the area of the CellTracker Blue-labeled protrusion-forming cell. We chose to do this because when infection foci are compared to one another within the same experimental group, the presence of larger or smaller cells could skew focus area determinations, even if the proportion of spreading bacteria is equivalent between different foci. By means of postacquisition thresholding and delineation of labeled L. monocytogenes, a mask encapsulating the bacteria associated with both the protrusion-forming and invagination-forming cells was obtained (Fig. 6G). We also delineated the area of the labeled protrusion-forming cell using a similar approach. We then determined the ratio of the area of infection to the area of the labeled protrusion-forming cell and found that it was reduced by about 40% when the invagination-forming cells were depleted of mDia1 (Fig. 6H).

Taken together, these findings point toward a key role for the accumulation of mDia1 and F-actin at L. monocytogenes membrane invaginations during bacterial cell-to-cell spreading events.

## DISCUSSION

Investigation into the mechanisms driving L. monocytogenes cell-to-cell spreading have historically (as well as recently) focused on processes that occur in the membrane protrusion-forming infected host cell, leaving the corresponding invagination-forming host cells poorly studied. Here, we set out to characterize the structure, composition, and role of the F-actin coat surrounding L. monocytogenes membrane invaginations. We show that these large caveolin-rich invaginations are surrounded by a shell of linear actin filaments that are polymerized by the formin mDia1. Crucially, bacterial intercellular spreading is significantly decreased when membrane invagination-forming host cells are depleted of mDia1.

Our finding that mDia1 is responsible for the generation of the actin shell that coats L. monocytogenes membrane invaginations represents a paradigm shift for three main reasons. First, it is well-known that the Arp2/3 complex and branched actin assembly are required throughout the L. monocytogenes infectious cycle of epithelial cells, including its initial entry into cells (12, 13, 16), evasion of host autophagic pathways (64–66), intracellular motility (16, 20–22, 49, 67), and cell-to-cell spreading (68, 69). It is thus unexpected that the formation and proper function of the caveolin-rich membrane invaginations mediating L. monocytogenes endocytosis rely exclusively on mDia1 and linear actin filaments. Of note, a previous study has suggested that L. monocytogenes exploits host formins during the formation of membrane protrusions, although formins are not essential and appear to cooperate with the Arp2/3 complex (47). Moreover, in that study, cell-to-cell spreading was reduced by almost 40% when mDia1 was silenced in both the protrusion-forming and invagination-forming host cells. Remarkably, the decrease in bacterial spreading that we report upon knockdown of mDia1 in only the invagination-forming host cells closely mimics the above findings. This suggests that mDia1 has a negligible role in the protrusion-forming host cells. This is in line with the fact that mDia1 does not localize to or affect the formation of comet/rocket tails (47). Furthermore, we could not detect a concentration of mDia1 at the L. monocytogenes-induced membrane protrusions that engaged neighboring cells. We conclude that the main role of mDia1 during bacterial cell-to-cell spreading is to optimize L. monocytogenes endocytosis by generating an actin-rich coat that keeps caveolin-1 in place at the invaginated host plasma membrane.

Second, our discovery that the actin shell at L. monocytogenes membrane invaginations consists of mDia1-dependent actin filaments provides the first example of the involvement of linear F-actin during the endocytosis of large cargoes. L. monocytogenes is not the only microbial pathogen that exploits formin proteins, as formins are acknowledged players during the Arp2/3 complex-dependent formation of membranous protrusions that allow the capture and entry of micro-sized particles such as bacteria from the extracellular milieu through phagocytosis and related processes (70–74). Plasma membrane invaginations leading to nano-sized spherical or tubular vesicles are commonly associated with branched actin arrays and the Arp2/3 complex, with the formation of clathrin-coated vesicles from both clathrin-coated pits (39, 40, 75; see also reference 2 for a review) and flat clathrin-coated plaques (41) providing the best-characterized examples. In addition, other endocytic pathways resulting in nano-sized vesicles such as fast endophilin-mediated endocytosis (FEME) and the CLIC/GEEC (clathrin-independent carriers [CLIC], glycosylphosphatidylinositol-anchored proteins [GPI-AP]-enriched early endosomal compartments [GEEC]) pathways also depend on actin polymerization by the Arp2/3 complex (see reference 2 for a review). It is only recently that evidence of a metazoan formin-dependent endocytic process has come to light. In hippocampal neurons, the formation of nano-sized synaptic vesicles occurs via a clathrin-independent and formin (mDia1)-dependent pathway (76). Thus, their and our findings suggest that not only the Arp2/3 complex but also formins can contribute to the formation of both nano- and micro-sized endocytic vesicles. This begs the question as to whether and how the linear actin filaments assembled by mDia1 power large micro-sized endocytic processes. It is also worth noting that L. monocytogenes membrane invaginations clearly differ from typical endocytic membrane invaginations as they are subjected to the pushing forces generated by the incoming bacterial membrane protrusions. Thus, such forces may replace those arisen from branched actin polymerization by the Arp2/3 complex, thus explaining its absence at the bacterial endocytic sites.

Finally, the physical link between caveolin-1 and linear actin filaments found at L. monocytogenes membrane invaginations has an unanticipated functional significance. Indeed, caveolae are tethered to stress fibers as part of actin-regulated mechanosensitive pathways (see reference 55 for a review). mDia1-dependent stress fibers are crucial for proper caveola organization and inward movement of the carriers as knockdown of mDia1 decreases the pool of stress fiber-coaligned caveolin-1 and leads to the formation of caveolin-1 clusters at the cell surface (77). In contrast, mDia1-mediated actin assembly is required to recruit and/or retain caveolin-1 at the invagination sites where L. monocytogenes-containing membrane protrusions are internalized. Thus, although L. monocytogenes membrane invaginations and caveolae share some molecular components (caveolin-1, Filamin A, Myo1c, and linear F-actin), they appear to be regulated by different mechanisms.

**FIG 7:**
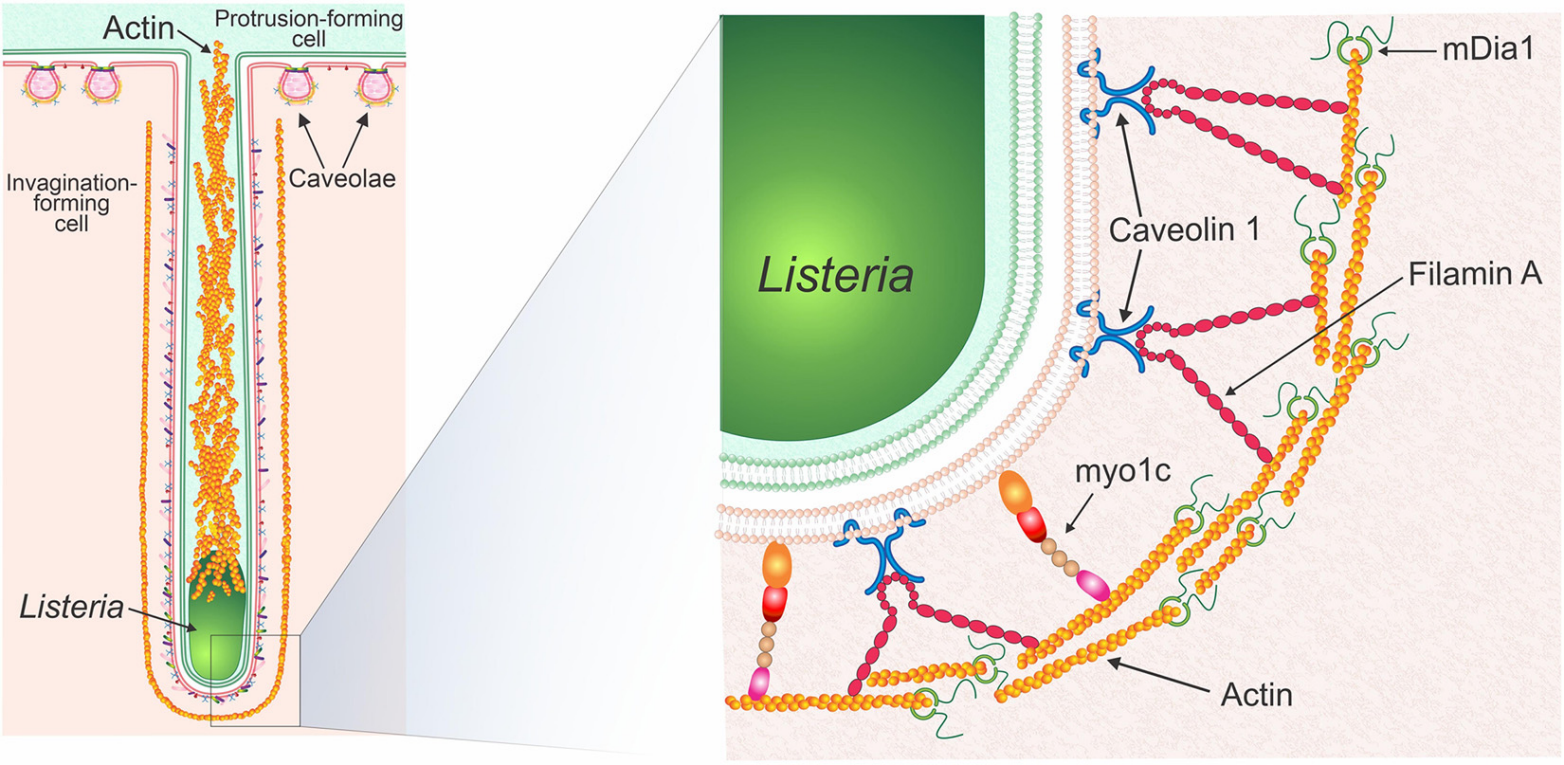
Proposed model of mDia1 and caveola-associated F-actin-binding proteins at L. monocytogenes membrane invaginations. Proposed model (modified from reference 26) demonstrating the organization of linear actin together with mDia1, Filamin A, and Myo1c. An L. monocytogenes membrane protrusion containing the bacteria together with its actin-rich tail is formed in the “green cell” (labeled “Protrusion-forming cell” in the model). The membrane protrusion is endocytosed by the “pink cell” (labeled “Invagination-forming cell” in the model) using a caveolin-based invagination. Linear actin filaments are depicted surrounding the membrane invagination with mDia1 nucleating and then growing the filaments. Filamin A and Myo1c link the parallel actin filaments to the plasma membrane of the invagination. Caveolae at the surface of the “pink cell” that are not exploited by the L. monocytogenes membrane protrusion/invagination are labeled in the model as “Caveolae.”

We propose a model of bacterial cell-to-cell spreading whereby a bacterial membrane protrusion utilizes actin filaments to push L. monocytogenes toward a neighboring host cell, thus deforming its cell surface. This sets in motion the formation of a bacterial membrane invagination enriched in caveolin-1 and its associated proteins to promote the endocytosis of the bacterium-containing membrane protrusion (25, 26). As actin is being polymerized, Filamin A and Myo1c are recruited to link the growing filaments to the membrane of the invagination (Fig. 7). In particular, Filamin A could directly link caveolin-1 (55, 56) found embedded within the membrane of the invaginations (26) to the underlying actin coat. Our finding that the loss of mDia1 and concomitant loss of Filamin A, F-actin, and caveolin at invaginations does not remove Myo1c suggests Myo1c could in fact bridge the thin filamentous actin coat and the lipid bilayer of the invagination itself by way of its N-terminal actin-binding motor domain (78) and C-terminal phosphatidylinositol-4,5-bisphosphate (PIP2)-binding pleckstrin homology (PH) domain (59). Indeed, our recent observation that phosphoinositides and phosphatidylserine cluster at L. monocytogenes invaginations (26) lends credence to this possibility. Furthermore, Filamin A along with α-actinin-1 and α-actinin-4, two other F-actin-cross-linking proteins that accumulate at L. monocytogenes membrane invaginations (36, 38), could impart additional rigidity (79) to the mDia1-nucleated actin coat and keep it localized close to the plasma membrane as the invagination deepens.

In summary, our work demonstrates that the assembly of a linear F-actin coat regulates the micro-sized caveolin-mediated endocytic events that allow L. monocytogenes to spread between host cells. This is the first evidence of linear actin filaments being used for the internalization of a caveolin-mediated endocytic structure and draws parallels to the branched actin networks that are assembled at sites of clathrin-mediated endocytosis. Most importantly, this work opens the door for investigations into the role of linear F-actin arrays at other large micro-sized internalization events as well as classical nano-sized caveolin-based endocytic processes.

## MATERIALS AND METHODS

### Cell culture.

Human cervical (HeLa) and placental (Jeg-3) epithelial cells were purchased from American Type Culture Collection (ATCC) (numbers CCL-2 and HTB-36, respectively). HeLa cell lines with stable knockdown of mDia1 (termed shmDia1) as well as the control [nontargeting] cells (termed control shRNA) were generated and characterized previously (45, 80). HeLa cells were cultured using Dulbecco’s modified Eagle’s medium (DMEM) containing high glucose (Gibco, Thermo Fisher Scientific) and supplemented with 10% fetal bovine serum (FBS) (Gibco, Thermo Fisher Scientific). The control shRNA and shmDia1 stable HeLa cell lines were also cultured at all times using media containing 2.5 μg/ml puromycin (MilliporeSigma). Jeg-3 cells were cultured using minimum essential medium (MEM) (Gibco, Thermo Fisher Scientific) supplemented with 10% FBS. The parental mouse embryonic fibroblast (MEF) cell line designated 10-4 was generated previously (48) and cultured using high-glucose DMEM containing 10% FBS and 2.0 μg/ml puromycin. To obtain Arpc2^−/−^ cells, parental 10-4 MEF cells were seeded into 10-cm culture dishes and then treated over the course of 3 days with 2.0 μg/ml puromycin (MilliporeSigma) and 2.0 μM 4-hydroxy-tamoxifen (4-OHT; MilliporeSigma) (or dimethyl sulfoxide [DMSO] for the controls) as described previously (48). All cell lines were maintained in a cell culture incubator at 37°C and 5% CO_2_. To seed cells for experiments, flasks containing cells were washed three times with Dulbecco’s phosphate-buffered saline without Ca^2+^ and Mg^2+^ (PBS [-/-]) (Gibco, Thermo Fisher Scientific), trypsinized with 0.05% trypsin-EDTA (Gibco, Thermo Fisher Scientific), counted, and seeded onto clear polystyrene 6-well or 24-well plates (Corning). For electron microscopy experiments, cells were seeded onto 24-well format 0.4-μm Transparent polyethylene terephthalate (PET) permeable cell culture inserts (Falcon).

### Bacterial strains and growth conditions.

The Listeria monocytogenes strain EGD BUG 600 was used throughout this study and grown at 37°C using either brain heart infusion (BHI) agar or broth (BD Biosciences).

### L. monocytogenes infections.

To infect cells, 2-ml overnight (12 to 16 h) shaken (at 225 rpm) broth cultures of L. monocytogenes were diluted 10-fold in fresh BHI broth (at a final volume of 10 ml) and then incubated at 37°C in a shaking incubator (at 225 rpm and approximately a 70° angle) until the culture reached an optical density reading of *A*_600_ of 1.00 (∼2 h of subculturing). At an *A*_600_ of 1.00, 1 ml of bacteria was centrifuged for 5 min at 9,600 × *g* (25°C). Prewarmed (37°C) 1× PBS [-/-] was then added to the pellet, and the bacteria were spun down for 5 min at 9,600 × *g*. PBS washing was repeated two additional times. After the final PBS wash, the pelleted bacteria were resuspended with 1 ml of prewarmed serum-free medium (DMEM or MEM; 37°C) and then diluted 100 to 1,000 times. The diluted bacteria were added onto culture plates containing host cells and incubated for at least 6 to 8 h to study actin comet/rocket tail and membrane protrusion/invagination formation.

### L. monocytogenes mixed-cell infections.

One batch of host cells was seeded at a density of 2 × 10^6^ per well in six-well format plates without coverslips. On the same day, in separate six-well plates (containing coverslips), a second batch of host cells was seeded at a density of 2.25 × 10^6^ per well. The following day, the host cells seeded previously at a density of 2.25 × 10^6^ were transfected (as described below) with DNA plasmids encoding the fluorescently tagged protein of interest to be examined at L. monocytogenes membrane invaginations. On day 3, the untransfected host cells (seeded previously at a density of 2 × 10^6^ per well) were infected (as described above) with wild-type L. monocytogenes at a multiplicity of infection (MOI) of ∼40. At 2 h postinfection, the infected cells were washed three times with PBS [-/-], and then 1 ml of prewarmed media containing 10% FBS and gentamicin (50 μg/ml) was added into each well to kill any remaining extracellular bacteria. After 3 h of infection, the infected cells were detached and enumerated as described previously (26, 81). Approximately 1 × 10^6^ infected cells were then overlaid onto well plates containing the previously uninfected/transfected cells. Gentamicin was added to a final concentration of 50 μg/ml. Samples were fixed 5 to 6 h following the overlaying procedure and stained as described below. The examination of fluorescently tagged proteins of interest at L. monocytogenes spreading events was performed by microscopic analysis of infection events where untransfected but infected cells were sending out L. monocytogenes membrane protrusions directly into (and generating invaginations within) the adjacent transfected cells (as visually determined by the microscopist through a combination of fluorescent and phase-contrast microscopy). Care was taken to ensure that each membrane protrusion could be visually traced back to the original sending (untransfected cell).

### L. monocytogenes mixed-cell spreading assay.

On day 1, control shRNA protrusion-forming cells (to be infected later with L. monocytogenes) were initially seeded into six-well format plastic plates (without glass coverslips) at a density of 3.0 × 10^5^. Also on day 1, 3.0 × 10^5^ control shRNA and shmDia1 invagination-forming cells were seeded into separate six-well format plastic plates (without glass coverslips). These numbers were selected so that the confluence of each well reached ∼100% on day 3 (the day of infection). On the day of the infections (2 days postseeding), culture plates containing the control shRNA protrusion-forming cells were rinsed five times with prewarmed PBS with Ca^2+^ and Mg^2+^ (PBS [+/+]), and then 0.8 ml of fresh prewarmed serum-free DMEM was added to each well. After this, 0.2 ml of the 1,000× diluted bacteria (as described above) was added to each well. The well plates were then spun down in a centrifuge for 2 min at 214 × *g* and then placed in a 37°C incubator for 2 h. After 2 h, the infected cells were washed three times with prewarmed PBS [+/+], and then 1 ml of DMEM containing 10% FBS, 20 μM CellTracker Blue (to label the cells), and 50 μg/ml gentamicin (to kill any extracellular bacteria) were added to each well. The plates were then incubated for 45 min in a 37°C incubator. After these 45 min, the now CellTracker Blue-labeled infected cells were rinsed five times with prewarmed PBS [+/+] and then detached from the wells. At the same time, the control shRNA and shmDia1 invagination-forming cells were also rinsed five times with prewarmed PBS [+/+] and then detached from the wells. Both cell populations were detached as described previously (26, 81). To the pelleted cells, 1 ml of fresh prewarmed DMEM containing 10% FBS and 50 μg/ml gentamicin (per well collected) was added in order to resuspend the cells. A ratio of 1:12.5 (protrusion-forming cells to invagination-forming cells) was used when combining the two cell populations. The following volume of cells was then added per well of a 24-well format plastic plate (containing glass coverslips and prefilled with 1 ml of DMEM containing 10% FBS and 50 μg/ml gentamicin): 10 μl of protrusion-forming cells and 135 μl of invagination-forming cells (either unlabeled control shRNA or shmDia1). After combining the two cell populations, we spun down the culture plates in a centrifuge for 2 min at 214 × *g* and then allowed the infections to proceed for 9 h in order to allow the bacteria to sufficiently spread out of the CellTracker Blue-labeled protrusion-forming cells into surrounding (and unlabeled) invagination-forming cells. After 9 h, the samples were fixed and permeabilized with 0.2% Triton X-100 in PBS [-/-] as described below. The L. monocytogenes bacteria were detected with rabbit anti-L. monocytogenes antibodies followed by secondary antibody labeling with Alexa Fluor 594-conjugated goat anti-rabbit antibodies. To determine the proportion of spreading bacteria per infection focus, the number of bacteria present within the confines of the invagination-forming cells was divided by the total number of bacteria contained within the entire infection focus. If required, additional fields of view were captured in order to enumerate all of the bacteria within a distinct infection focus. To determine the number of infected host cells per infection focus, host cells infected with bacteria were manually enumerated. This was accomplished using a combination of (i) examining bacterial actin-rich structures (actin clouds, comet/rocket tails, and membrane protrusions) generated by the bacteria within the host cells and (ii) general visual detection of distinct host cells by their cell borders (via F-actin staining) and nuclei (via phase-contrast microscopy). Determination of the ratio of the area of infection to the area of the protrusion-forming cell was performed based on the methods described by Talman and colleagues (82).

### Reagents and antibodies.

Antibodies and reagents used in this study included the following: Alexa Fluor 594- and 488-conjugated phalloidin (Invitrogen), CellTracker Blue (20 μM for mixed population spreading assay; Invitrogen), Alexa Fluor 594- and 488-conjugated goat anti-rabbit and goat anti-mouse antibodies (2 μg/ml; Invitrogen), rabbit anti-mDia1 (2.07 μg/ml for immunofluorescence; Proteintech Group, 20624-1-AP [human targeting]), rabbit anti-p34 (10 μg/ml for immunofluorescence; MilliporeSigma, 07-227-I-100UG), mouse anti-CD147 (10 μg/ml for immunofluorescence; Abcam, ab666), rabbit anti-caveolin-1 (1 μg/ml for Western blot; Abcam, ab2910), mouse anti-α-tubulin (1:1,000 for Western blot; Developmental Studies Hybridoma Bank, 12G10), rabbit anti-L. monocytogenes (1:300 for immunofluorescence,; BD Difco, 223021), and horseradish peroxidase (HRP)-conjugated goat anti-mouse and goat anti-rabbit antibodies (1 μg/ml; Invitrogen).

### Immunolocalization.

Cells on glass coverslips were fixed at room temperature (in the dark) for 15 min using prewarmed (37°C) 3% paraformaldehyde (prepared in 150 mM NaCl, 4 mM Na/K PO_4_, 5.0 mM KCl [pH 7.3]) and then washed three times using PBS [-/-]. Cells were permeabilized using room temperature 0.2% Triton X-100 (prepared in PBS [-/-]) for 5 min or −20°C acetone for 10 min. Following Triton X-100 permeabilization, coverslips were rinsed three times with PBS [-/-], whereas acetone-treated coverslips were left to dry at room temperature for 30 min. All samples were blocked with 5% normal goat serum {prepared in TPBS/BSA (PBS [-/-], 0.5% Tween 20, and 0.1% bovine serum albumin [BSA]} for 25 min and then incubated overnight at 4°C with primary antibodies also prepared in TPBS/BSA. The next day, coverslips were washed three times with TPBS/BSA for 10 min and then treated with secondary antibodies (Alexa Fluor 594- or 488-conjugated goat anti-rabbit or goat anti-mouse; prepared in TPBS/BSA) at room temperature (in the dark) for 2 h. To visualize F-actin, samples were treated with Alexa Fluor 594-or 488-conjugated phalloidin (prepared in PBS [-/-]) for 10 min. Samples were washed three times with PBS [-/-] and mounted onto glass microscope slides using ProLong glass antifade mountant with NucBlue STain (P36981; Invitrogen).

### Lysate preparation and Western blotting.

Cells were washed five times with prewarmed PBS [+/+] and then treated on ice for 5 min with prechilled (4°C) radioimmunoprecipitation assay (RIPA) lysis buffer (150 mM NaCl, 50 mM Tris [pH 7.4], 5 mM EDTA, 1% Nonidet P-40, 1% deoxycholic acid, 10% sodium dodecyl sulfate [SDS]) containing the cOmplete Mini EDTA-free protease inhibitor cocktail (Roche). Cell scrapers were used to help disrupt the cells. The cell lysates were then collected into prechilled (4°C) microcentrifuge tubes. The lysates were spun at 4°C and 10,000 × *g* for 10 min to pellet any cellular debris and nucleic acids; the supernatants were collected into fresh prechilled (4°C) microcentrifuge tubes, flash frozen using liquid nitrogen, and then immediately stored in a −80°C freezer. Protein concentrations were determined using a bicinchoninic acid (BCA) assay kit (Pierce). For Western blotting, lysate samples were prepared using 6× Laemmli buffer and then boiled (at 100°C) for 10 min. Equal amounts of protein were loaded onto 10% SDS-polyacrylamide gels and resolved by electrophoresis. Gels were rinsed in distilled water for 5 min and then transferred onto nitrocellulose membranes using a Trans-Blot SD semidry transfer cell (Bio-Rad). Membranes were washed for 5 min in TBST (Tris-buffered saline, 0.05% Tween 20) with shaking, blocked with 4% Blotto (Santa Cruz Biotechnology) prepared in TBST (1-h shaking), and then treated with primary antibodies (diluted in TBST containing 1% BSA) overnight at 4°C. The next day, membranes were rinsed three times with TBST for 10 min and then three times with TBST for 5 min prior to incubation with secondary antibodies (HRP-conjugated goat anti-rabbit or goat anti-mouse) for 1 h at room temperature. To visualize protein bands, membranes were treated with Western Lightning Plus-ECL (PerkinElmer) following the manufacturer’s instructions imaged using a Fujifilm LAS-4000 imager (Fujifilm). To confirm equivalent loading, membranes were stripped using mild stripping buffer (1.5% glycine, 0.1% SDS, 1% Tween 20 [pH 2.2]) and reprobed using the mouse anti-α-tubulin targeting antibody.

### Western blot quantification.

Protein quantification was performed using the 16-bit image files and the “gels” tool in ImageJ. All analyzed lanes were first adjusted for loading by normalizing the loading control signal of each lane against the signal of a randomly selected lane (“lane *x*”). Following this, the mDia1 signal for each lane was also normalized against the mDia1 signal of “lane *x*.” To obtain the adjusted and relative mDia1 protein levels of each lane, the relative mDia1 signal of each lane was divided by the relative loading control of that same lane.

### DNA constructs.

Plasmids containing mEmerald-mDia1, mEmerald-ARPp34, mEmerald-Filamin A, mCherry-Caveolin-1 (83), mKate-LifeAct, eGFP-LifeAct (gifts from Michael Davidson; plasmid 54156, plasmid 53997, plasmid 54098, plasmid 55008, plasmids 54697 and 54610, respectively) were obtained from Addgene. The plasmid encoding CD147-GFP was generated previously (84). The plasmid encoding Myo1c-GFP was obtained from Ana-Maria Lennon-Duménil (acquired via Kristine Schauer).

### Cell culture transfections.

All DNA transfections of cultured cells were performed using the jetPEI (for HeLa and Jeg-3 cells) or jetPRIME (for MEF cells) transfection reagents (Polyplus Transfection) and carried out according to the manufacturer’s instructions. Briefly, the prepared DNA transfection reagent mixture was pipetted dropwise onto cultured cells contained in six-well format plates. The cells were then placed inside an incubator (37°C) for 4 h. Following this, the culture medium was replaced, and cells were incubated for an additional 24 h at 37°C to allow for sufficient expression of the transfected plasmid cDNA product.

### Electron microscopy.

For standard electron microscopy, Jeg-3 cells were grown on transwell membranes (Falcon, product number 353095; Corning, USA) placed within 24-well format plastic cell culture plates (Costar, product number 3524; Corning, USA). Medium was replaced with fixative (1.5% paraformaldehyde, 1.5% glutaraldehyde, 0.1 M sodium cacodylate [pH 7.3] at room temperature [rt]) for 2 to 3 h, and then the fixative was replaced with buffer (0.1 M sodium cacodylate [pH 7.3] [rt]). The membranes, with attached cells, were then cut into pieces approximately 1.5 cm by 1.5 cm. The pieces were placed in glass vials and washed two times (10 min per wash) with fresh buffer. The samples were postfixed for 1 h on ice with 1% osmium tetroxide in 0.1 M sodium cacodylate (pH 7.3). The samples were washed three times (10 min each wash) with double-distilled water (ddH_2_O) (rt) and then stained *en bloc* with 1% aqueous uranyl acetate (rt). The membranes were again washed three times with ddH2O (rt) and then dehydrated through an ascending series of ethyl alcohols (30%, 50%, 70%, 95%, and 100% twice [10 min each time]) followed by two treatments with propylene oxide (15 min each). The samples were then placed in 1:1 propylene oxide-EMBED 812 resin (Electron Microscopy Sciences, Hatfield, PA) and left overnight. The next day, the membranes were passed through two changes of 100% EMBED 812 resin and then placed on glass slides with the cells face up. Embedding capsules were filled with resin and inverted onto the membranes, and then the slides with membranes and embedding capsules were placed in an oven (60°C) for the resin to polymerize for 48 h. After polymerization, the capsules (with embedded cells) were carefully separated from the membranes that remained attached to the slides. The cell layers at the surfaces of the block were sectioned en face using a Leica EM UC6 ultramicrotome fitted with Ultra-AMF 3.00-mm diamond knife (DiATOME) and collected onto copper grids. The sections were stained with uranyl acetate and lead citrate and then imaged using a Talos L120C transmission electron microscope (Thermo Fisher Scientific) operated at 120 kV.

### S1 decoration.

For extraction and S1 decoration, Jeg-3 cells were grown on transwell membranes (Falcon, product number 353095; Corning, USA) placed within 24-well format plastic cell culture plates (Costar, product number 3524; Corning, USA). Cells were briefly washed with suspension buffer (10 mM Na phosphate, 135 mM NaCl, 5 mM MgCl, 2 mM EDTA [pH 7.0]) and then extracted with the buffer containing 5% (wt/vol) glycerol for 1 min (rt). The extraction buffer was replaced with 2 mg/ml S1 (made up by diluting the stock solution of S1 [3.3 mg/ml of rabbit psoas S1 myosin; catalog no. CS-MYS04, Cytoskeleton, Inc., USA] with suspension buffer) and the cells incubated (rt) for 15 min. Cells were briefly washed with buffer and then immediately fixed in 10 mM Na phosphate buffer containing 1% glutaraldehyde and 0.2% tannic acid (Mallinckrodt) (pH 7.0) for 30 min (rt). The fixative was replaced with 10 mM phosphate buffer, and then the membranes were cut from the transwells, placed in glass vials, and stored overnight (rt). The membranes were washed two times with 10 mM Na phosphate buffer (10 min per wash) and then postfixed for 1 h on ice in buffer containing 1% OsO4. The membranes were washed three times with ddH_2_O (rt) and then postfixed, *en bloc* stained, dehydrated, and infiltrated with resin as indicated above. The membranes were placed on flat silicon supports with the cells face up. Embedding capsules were filled with resin, inverted onto the membranes, and then placed in an oven (60°C) to polymerize. After polymerization, the capsules with membranes still attached were separated from the silicon supports, and the blocks were trimmed. Blocks were sectioned through the membrane until the cell layer was reached, and then sections were collected, stained, and imaged as indicated above.

### Immunofluorescence microscopy.

A Leica DMI4000B (Leica Microsystems) inverted fluorescence microscope equipped with a Hamamatsu Orca R2 charge-coupled-device (CCD) camera (Hamamatsu Photonics) was used to acquire all immunofluorescent images. All devices were controlled by the MetaMorph Imaging System software (Universal Imaging). All images were acquired using a 100× oil immersion objective (×1,000 magnification overall). Images were evaluated and processed using Metamorph Imaging System software or ImageJ. The pixel intensity plots (line scan analyses) were performed using ImageJ by the microscopist whereby the line tool was used to first draw a 1.5- to 2.5-μm line perpendicularly across the structures. Following this, the “plot profile” tool was used to obtain the pixel intensity value (from 0 to 255) of the protein of interest and actin. Lines were excluded or shifted if intense signal from cellular structures (such as stress fibers), random artifacts, or other nearby invaginations interfered with the profile of interest. Line scan analyses were replicated at least three times (and up to six times) for each protein examined. Heat maps of the representative images were generated by applying the “inferno” LUT (look up table) to the corresponding 8-bit image file using ImageJ. In these heat maps, intensity values range linearly from 0 (low) to 255 (high), with the highest level of protein signal represented as yellow. The signal intensity then decreases successively from orange to red then purple and finally black (a pixel intensity of 0).

### Statistical analysis.

Statistical analysis was performed using GraphPad Prism version 6.01. All results involving immunofluorescence microscopy, line scan analyses, and Western blotting were obtained from experiments performed at least three times (*n* = 3). All presented images are representative of the experiments performed. See also the corresponding figure legends for information regarding the quantification of protein localization frequency at membrane invaginations, Western blotting (protein levels), bacterial spreading assays (the exact number of bacteria enumerated and samples/field of views analyzed), and whether or not measurements were normalized to controls. For all quantified data, the statistical tests utilized and accompanying *P* values can also be found in the corresponding figure legends.
